# Self-aggregating long-acting injectable microcrystals

**DOI:** 10.1038/s44286-025-00194-x

**Published:** 2025-03-24

**Authors:** Vivian R. Feig, Sanghyun Park, Pier Giuseppe Rivano, Jinhee Kim, Benjamin Muller, Ashka Patel, Caroline Dial, Sofia Gonzalez, Hannah Carlisle, Flavia Codreanu, Aaron Lopes, Ayten E. Erdogan, Niora Fabian, Ashley Guevara, Andrew Pettinari, Jason Li, Jia Liang, Gary W. Liu, Mark W. Tibbitt, Giovanni Traverso

**Affiliations:** 1 Division of Gastroenterology, Department of Medicine, Brigham and Women’s Hospital, Harvard Medical School, Boston, MA USA; 2 Koch Institute for Integrative Cancer Research, Massachusetts Institute of Technology, Cambridge, MA USA; 3 Department of Mechanical Engineering, Stanford University, Stanford, CA USA; 4 Department of Mechanical Engineering, Massachusetts Institute of Technology, Boston, MA USA; 5 Macromolecular Engineering Laboratory, ETH Zurich, Zurich, Switzerland; 6 Department of Pharmacology and Toxicology, University of Toronto, Toronto, Ontario Canada; 7 Department of Bioengineering, Northeastern University, Boston, MA USA; 8 Behavioral Neuroscience Program, College of Science, Northeastern University, Boston, MA USA; 9 Department of Chemistry, Northeastern University, Boston, MA USA; 10 Department of Chemistry, University of Toronto, Toronto, Ontario Canada; 11 Division of Comparative Medicine, Massachusetts Institute of Technology, Cambridge, MA USA; 12 Broad Institute of MIT and Harvard, Cambridge, MA USA

**Keywords:** Materials for devices, Drug delivery

## Abstract

Injectable drug depots have transformed our capacity to enhance medication adherence through dose simplification. Central to patient adoption of injectables is the acceptability of needle injections, with needle gauge as a key factor informing patient discomfort. Maximizing drug loading in injectables supports longer drug release while reducing injection volume and discomfort. Here, to address these requirements, we developed self-aggregating long-acting injectable microcrystals (SLIM), an injectable formulation containing drug microcrystals that self-aggregate in the subcutaneous space to form a monolithic implant with a low ratio of polymer excipient to drug (0.0625:1 w/w). By minimizing polymer content, SLIM supports injection through low-profile needles (<25 G) with high drug loading (293 mg ml^−1^). We demonstrate in vitro and in vivo that self-aggregation is driven by solvent exchange at the injection site and that slower-exchanging solvents result in increased microcrystal compaction and reduced implant porosity. We further show that self-aggregation enhances long-term drug release in rodents. We anticipate that SLIM could enable low-cost interventions for contraceptives.

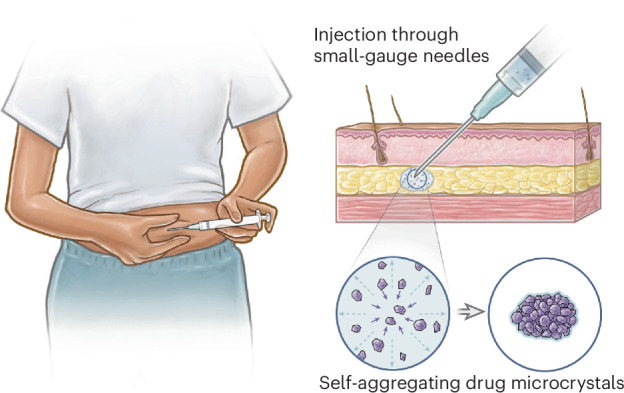

## Abstract (web summary)

This study reports on self-aggregating injectable microcrystals for administering long-acting drug implants via low-profile needles, a key factor in patient adoption. Microcrystal self-aggregation is engineered through a solvent exchange process to form depots with minimal polymer excipient, demonstrating enhanced long-term release of a model contraceptive drug in rodents.

## Main

**Fig. 1:**
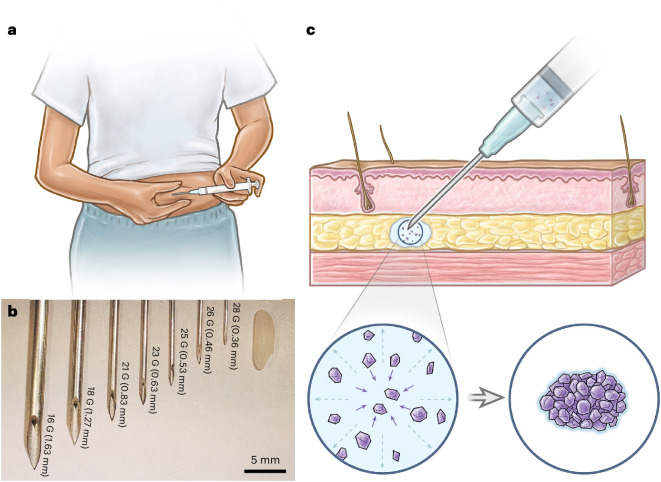
Overview of SLIM. **a**, Schematic of the self-injection procedure. **b**, An image of different needle gauges ranging from 18 G to 28 G, compared against a grain of rice. **c**, Schematic of subcutaneous environment highlighting the solvent-exchange-driven self-aggregation of microcrystals into a compacted implant. Illustrations in **a** and **c** by Virginia E. Fulford, Alar Illustration.

Long-acting drug delivery systems that can be self-administered via subcutaneous injections are highly desired. Such technologies combine the long-term drug release of surgically administered implant systems, which improve patient compliance by eliminating the need to remember to take a pill every day, with the ease of administration of injectables, which is particularly important for patients in low-resource settings without easy access to medical infrastructure (Fig. 1a)^1^. Needle size is a crucial consideration for the commercial translation of subcutaneous injectables: patient acceptance of therapeutics increases as needle size decreases, which is probably related to how smaller needles tend to cause less bruising or bleeding at the injection site (Fig. 1b)^2,3^. Furthermore, self-administration is possible only for relatively low formulation viscosities because patients can only comfortably apply a maximum of 64 N of force to a syringe by hand^4^. So far, however, it has been challenging for most marketed long-acting injectables to combine long durations of action (>3 months) with the capacity for self-administration through small-gauge needles as they rely on polymer excipients to sustain long-term drug release and secure mechanical integrity, which often substantially increase solution viscosity at required concentrations.

Existing long-acting injectables can be divided into two categories: microparticle suspensions and in situ forming implants (ISFIs)^2^. Microparticle suspensions leverage sustained release from micrometer-sized (1–1,000 μm) particles encapsulating solid or liquid drug substances within a polymeric matrix, whereas ISFIs are liquid or semisolid formulations that transform into solid-like structures at the target site. These systems release drugs through various mechanisms, including polymer degradation, cross-linking, phase separation via solvent exchange, and self-assembly into hydrogels^5,6^. In both types of formulations, it is challenging to combine effective long-term release with injectability through small-gauge needles compatible with self-administration, a problem that is particularly relevant for low-potency drugs (Supplementary Tables 1 and 2)^7–10^. While microparticle suspensions can achieve long release durations using slowly degrading polymers such as polylactic acid or polycaprolactone (PCL), their relatively low drug loading (% w/w) remains a potential concern for long-acting applications. Increasing particle size hampers injectability through smaller needles owing to clogging. Furthermore, the inability to easily retrieve these microspheres once administered poses challenges in scenarios where reversibility is required, such as removing contraceptive drugs to enable a return to fertility. Meanwhile, most previously reported ISFIs utilize formulations with high ratios of polymer to drug (typically >1:1 w/w), and increasing formulation concentration to increase drug loading reduces injectability because viscosity increases dramatically with higher concentrations of polymer excipients^11^. Indeed, the only currently US Food and Drug Administration-approved ISFIs (Atrigel and its derivatives, including Eligard, Sublocade and Perseris) all require administration through large 18–20 G needles (Supplementary Table 2)^6^.

Injectables in the form of micro/nanocrystal drug suspensions could circumvent the aforementioned challenges because they do not depend on polymeric excipients to modulate release. Instead, drugs are released through the surface-based erosion of each crystal^12^. Surface erosion can translate into markedly extended release times for poorly soluble drugs as compared with solubilized compounds. However, most drug crystal suspensions are aqueous formulations with limited duration of release and poor consistency because of their high surface area and the fact that their release kinetics depend on the crystal size distribution, which is difficult to control^13,14^. As with microparticle suspensions, drug crystal suspensions also cannot be easily retrieved after administration.

Although these challenges are applicable to most long-acting injectables, we focus on their unmet needs for contraception. A long-acting contraceptive implant that can be self-administered via injection would be a much-needed addition to the current suite of family planning options available to women, especially for people in low-resource settings where options for contraception and health care facilities are limited^15^. Currently, the only self-administrable commercialized contraceptive injectables are Depo-Provera and Sayana Press, which are both aqueous microcrystal suspensions that can be injected through moderately small needles (23 G and 26 G) because of the absence of polymer excipient^16,17^. However, compared with the surgically inserted 1.5-year-long contraceptive implant Nexplanon^18^, their duration of action is limited to only 3 months potentially because of the high surface area of the injected drug crystals.

To address the key challenges in self-administrable long-acting injectables, we designed self-aggregating long-acting injectable microcrystals (SLIM) for the contraceptive drug levonorgestrel (LNG), a progestin drug with a partition coefficient (log*P*) of 3.8 that is poorly soluble in aqueous environments^19,20^. The SLIM system has the following characteristics: (1) high drug loading (293 mg ml^−1^) with a low polymer-to-drug ratio over an order of magnitude lower than previous ISFIs (0.0625:1 w/w), (2) sufficiently low formulation viscosity to support injection forces <64 N through a 25 G needle and (3) the ability to self-aggregate into a solid implant in situ in the subcutaneous space with sufficient mechanical robustness and low surface area to enable sustained release over 3 months. This combination of properties is achieved by driving the self-aggregation of solid LNG microcrystals, with an average diameter of 2–3 μm (Supplementary Fig. 1), from suspension into a monolithic and compacted implant. Consistent with other granular systems comprising micrometer-scale particle building blocks, we propose that contact forces between compacted microcrystals are sufficient to enable the aggregated implant to behave like a monolithic solid (Fig. 1c)^21^. Furthermore, because LNG microcrystals are water-impermeable solids, LNG molecules are released into surrounding media via surface erosion (Supplementary Fig. 2). Without accounting for water infiltration into the aggregated implant, increasing the packing density of the LNG microcrystals is expected to reduce the average flux of active drug species from the implant and, thus, extend the total release time by reducing the outer surface area (Supplementary Note 1 and Supplementary Fig. 3). This effect is expected to be further magnified by the fact that increasing compaction should also reduce water infiltration into the bulk of the implant, which should increasingly confine drug release to the outer implant surface^7^.

## Results

### In situ self-aggregating LNG microcrystals via solvent exchange

We hypothesized that in situ self-aggregation of compacted LNG microcrystals could be achieved by engineering the exchange rate between the solvent of the drug suspension and the aqueous surroundings of subcutaneous tissue. While self-aggregation of microparticles upon solvent evaporation and precipitation of solid crystals from solution upon liquid–liquid solvent exchange have both been studied before in the literature, we focus here on the self-aggregation of preformed microcrystals within a suspension during liquid–liquid exchange^22,23^.

**Fig. 2:**
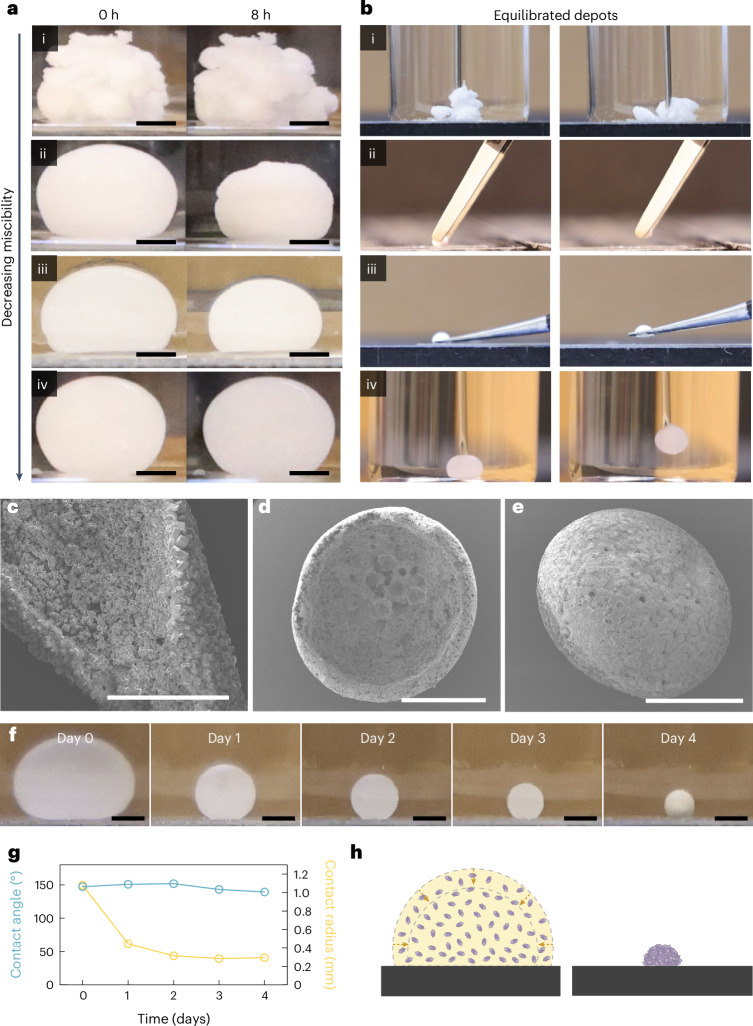
In situ self-aggregation of drug crystals via solvent exchange. **a**,**b**, A visual representation of the effect of three different solvents with different miscibility with the aqueous exchange medium: (i) NMP in PBS (perfectly miscible), (ii) BA (partially miscible) in PBS, (iii) BB in PBS with 10% SDS (partially miscible) and (iv) BB in PBS (immiscible). The figure illustrates the effect of the three solvents on the self-aggregation dynamics (**a**) and on the mechanical integrity of the equilibrated depots (**b**). Scale bars, 1 mm. **c**, Self-supported concave structure observed for 50 mg ml^−1^ of LNG in BA. Scale bar, 300 μm. **d**, Enhanced self-supported concave structure observed for 50 mg ml^−1^ of LNG in BA with small addition of PCL. Scale bar, 1 mm. **e**, No concave structure observed for slower-exchanging system of 50 mg ml^−1^ LNG in BB with PBS/5 wt.% SDS. Scale bar, 500 μm. **f**,**g**, Droplets of 50 mg ml^−1^ LNG in BB shrank in CCA mode over 4 days in PBS with 5% SDS (**f**), with an approximately constant contact angle and a continually shrinking contact radius (**g**). Scale bars, 1 mm. **h**, Schematics illustrating the compaction of LNG crystals into a dense sphere as a result of droplet shrinking with an unpinned contact line. Source data

An in vitro method was developed to study the impact of four different solvent exchange rates on the three-dimensional (3D) self-aggregation of LNG microcrystals upon injection into a medium of phosphate-buffered saline (PBS, pH 7.4) (Fig. 2 and Supplementary Fig. 4). Three solvents, previously used in injectable formulations in humans and encompassing a range of levels of miscibility with water, were identified^24,25^: *N*-methyl-2-pyrrolidone (NMP) (fully miscible), benzyl alcohol (BA) (partially miscible) and benzyl benzoate (BB) (immiscible). The rate of BB solvent exchange could be accelerated by adding surfactant molecules into the surrounding PBS medium (Supplementary Fig. 5). An intermediate solvent exchange rate between BA in PBS and BB in PBS was therefore realized by injecting BB formulations into PBS medium containing sodium dodecyl sulfate (SDS).

The rate and magnitude with which formulations comprising 293 mg ml^−1^ LNG shrank in volume upon injection into exchange media depended on the solvent–medium miscibility. The LNG–NMP formulation formed an equilibrated loose solid rapidly, with no further volume change observed after injection (Fig. 2a(i)). Rapid exchange between NMP and PBS inhibited the formation of a liquid–liquid interface, such that the resulting depot possessed a fluffy appearance with mechanical properties too weak to be grasped by tweezers (Fig. 2b(i)). At the other extreme, LNG–BB formulation injected into PBS also did not exhibit any volume change (Fig. 2a(iv)) and therefore did not solidify (Fig. 2b(iv)), although a clear liquid–liquid interface between the formulation and the exchange medium was observed.

Meanwhile, depot solidification was observed for the intermediate solvent exchange rates, with the depot volume equilibrating fastest for LNG–BA in PBS (Fig. 2a(ii)), followed by LNG–BB in PBS medium containing 10 wt.% SDS (PBS/10 wt.% SDS) (Fig. 2a(iii)) and LNG–BB in PBS/5 wt.% SDS. At completion, all depots formed from these intermediate exchange rates were solid enough to be handled by tweezers without fracturing (Fig. 2b(ii,iii)). Interestingly, when suspension concentrations were reduced to 50 mg ml^−1^ LNG, a self-supported concave structure was observed for depots aggregated from LNG–BA in PBS (Fig. 2c and Supplementary Fig. 6); a concave dome appearance was even more evident when the formulation was coformulated with PCL (Fig. 2d), a commonly used polymer excipient that dissolves in BA and undergoes phase inversion to solidify upon solvent exchange. These structures indicate that particle–particle interactions are sufficiently strong to support the weight of the structure, against the counteracting force of gravity. Meanwhile, no concavity was observed in depots formed from the slower-exchanging systems of 50 mg ml^−1^ LNG in BB with PBS/5 wt.% SDS (Fig. 2e) and PBS/10 wt.% SDS (Supplementary Fig. 7), which resulted in densely compacted LNG spheroids.

Based on these observations, we postulate that self-aggregation of LNG microcrystals via exchange between partially miscible solvents is analogous to aggregation of colloidal particles in an evaporating sessile droplet, a scenario that has so far been more thoroughly explored in the scientific literature^26,27^. Despite our system involving a liquid–liquid interface rather than a liquid–air one, both systems share the presence of a distinct interface between two phases and the removal of the liquid carrier through the interface. Just as evaporation rate can be modulated by temperature or relative humidity, we modulate solvent exchange rate by changing the miscibility of the solvent with its aqueous surroundings. In evaporating colloidal sessile droplets, the final morphology of the aggregated particles depends on whether the contact line is pinned during evaporation, which affects whether the droplet shrinks with a constant contact angle (CCA) or constant contact radius (CCR). In CCA mode, the contact line is freely moving and the contact angle remains constant, whereas in CCR mode the contact line is pinned and the wetted substrate area remains constant^26^. In our slower-exchanging LNG–BB systems in PBS with SDS, the drug-loaded formulation droplet shrank in CCA mode: the contact radius steadily decreased throughout the course of the depot formation period, while the contact angle remained steady above 90° (Fig. 2f,g, LNG–BB in PBS/5% SDS). In CCA mode, as the contact line shrinks radially inward, particles are driven to aggregate toward the center as well^28^; this is consistent with our forming compact LNG spheres from the LNG–BB and PBS–SDS systems (Fig. 2h).

While the LNG–BA contact angle is also greater than 90°, the contrast between depots formed from LNG–BA with PBS and LNG–BB with PBS and SDS can be understood by analogy to the effect of substrate heating in high-contact-angle evaporating droplets. In the latter case, the particles are transported toward the contact line by the radial outward capillary flow and away from the contact line by the Marangoni flow induced by thermal gradients at the liquid–air interface generating a stagnation region^28^. This stagnation region of particles at the contact line leads to ‘self-pinning’, a phenomenon denoted by Deegan^29^ that inhibits the droplet to shrink by CCA and favoring CCR instead (Supplementary Fig. 8). We hypothesize that, in our system, when faster solvent exchange occurs, Marangoni flows may be similarly enhanced by increased surface tension gradients caused by differences in diffusion rate and different LNG and/or solute concentration along the liquid–liquid interface. The concave dome structures observed from LNG–BA may arise from this self-pinning effect combined with interparticle jamming, which reinforces the temporary structures and prevents complete compaction into spherical depots. A more detailed mechanistic investigation will be required to fully elucidate these interactions in future studies.

Precipitation of dissolved LNG from the suspension, which is governed by secondary nucleation and growth, may also contribute to the final depot structure by increasing the overall packing density^30^. Consistent with this, the sizes of individual LNG crystals increased over time during solvent exchange between LNG–BA and PBS (Supplementary Fig. 9). Powder X-ray diffraction (XRD) analysis confirmed that all newly precipitated LNG in depots formed from suspensions via intermediate solvent exchange rates (LNG–BA in PBS and LNG–BB in PBS/10 wt.% SDS) shared the same crystalline structure as the initial microcrystals (Supplementary Fig. 10a,b). Meanwhile, the XRD spectrum of samples obtained from solvent exchange between LNG–NMP and PBS showed two peaks distinct from as-received LNG, suggesting the presence of an additional crystalline polymorph (Supplementary Fig. 10c). Similarly, LNG–BA crystals derived through primary nucleation and growth from a solution where no LNG microcrystals were initially present also resulted in diffraction peaks distinct from those of the original LNG (Supplementary Fig. 10d). These results suggest that distinct crystalline polymorphs arise when local concentrations of dissolved LNG exceed their saturation threshold without being sufficiently close to preexisting microcrystals, which may occur when the solvent is too highly miscible with the surrounding medium.

Altogether, moderate solvent–medium miscibility and poor substrate wetting results in a gradually receding liquid–liquid interface that drives the formation of a solidified monolithic implant in vitro. Within subcutaneous tissue in vivo, we anticipate that both these factors are ultimately dictated by the miscibility between the solvent and aqueous media, as the droplet will be fully surrounded by water-laden tissue. An LNG formulation comprising solvent that is moderately miscible with saline should therefore still be expected to freely shrink in vivo without needing to account for interfacial pinning. For evaporating droplets, the CCA mode is essentially identical to evaporation in freely suspended drops^26^; therefore, we anticipate that formulations with moderate solvent–saline miscibility should also enable LNG compaction to occur in vivo. Finally, moderate miscibility also helps to ensure that new LNG solids that precipitate during the solvent exchange process are morphologically identical to the preexisting LNG microcrystals.

### Development of a biocompatible LNG-based SLIM formulation

Our in vitro results suggest that a suspension drug formulation comprising a solvent that exchanges slowly with its surroundings is key to the self-aggregation of the drug particles into a monolithic implant. To apply these findings to a formulation that can be used *in vivo*, we next assessed the biocompatibility of the different solvents as well as their in vivo solvent exchange rates within subcutaneous tissue, which we expected may be different from our in vitro model.

NMP, BA and BB have all been previously utilized in subcutaneous drug formulations, with BA and BB typically being used in small quantities as additives^24,25^. Consequently, before testing any formulations comprising BA or BB as the sole solvents, we first investigated their acute and chronic toxicity after a single subcutaneous injection in rats. The solvents were administered alone at either a low dose (240 µl kg^−1^ body weight) or a high dose (1,600 µl kg^−1^ body weight). Rats were euthanized at either 3 days or 28 days post-injection to histologically evaluate the acute and chronic inflammatory responses, respectively. Although BB induced local inflammation at the injection at the high dose, no evidence of systemic toxicity was observed (Supplementary Figs. 11 and 12). At the low dose, BB did not show any noticeable acute or chronic inflammation. By contrast, rats injected with BA at the high dose exhibited severe morbidity within 2 h and were subsequently euthanized. Furthermore, even at the low dose, BA induced some visible bruising and inflammation at the injection site. Consequently, we decided to exclude BA from further studies.

**Fig. 3:**
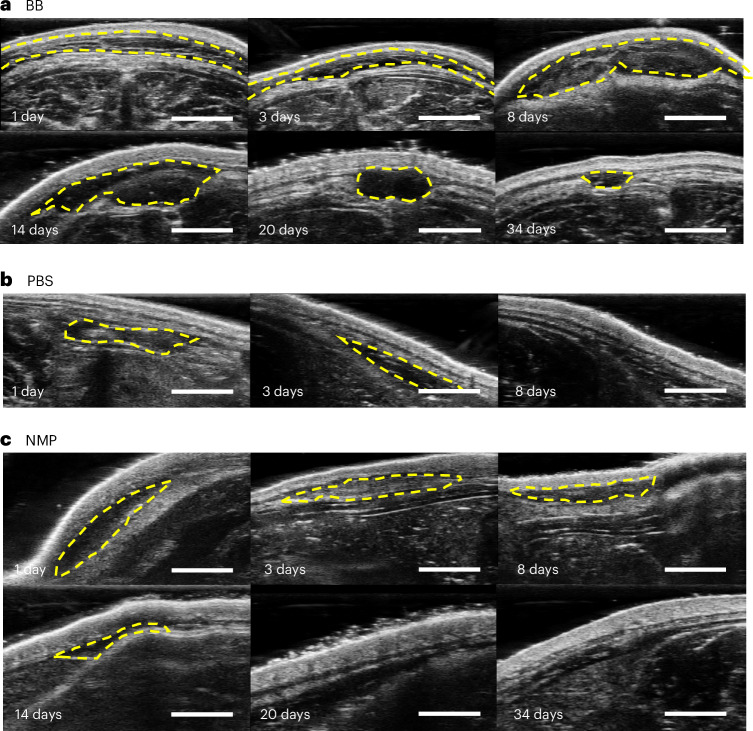
Assessment of solvent exchange in vivo*.* **a**–**c**, Ultrasound images depicting the clearance of BB within subcutaneous tissue over 34 days (**a**), PBS clearance over 8 days (**b**) and NMP clearance over 20 days (**c**). Yellow dashed circles indicate the contour of the injected solvent. Scale bars, 5 mm.

With the selected biocompatible solvents, we next examined their relative rates of solvent exchange with the subcutaneous environment by observing their disappearance rates locally from the injection site, with a bolus injection of PBS as the control group. For this, 0.5 ml of each solvent was subcutaneously injected, and their clearance locally from the injection site was monitored via ultrasound imaging. We observed that the BB bolus disappeared from the injection site within around 37 days (Fig. 3a). Whereas BB was found to be completely immiscible with PBS in vitro, subcutaneous tissue by contrast possesses fatty and lipophilic components that probably facilitate clearance, analogous to how adding surfactant into the exchange medium in vitro also accelerated BB exchange with its surroundings. By contrast, PBS and NMP cleared at much faster rates than BB (3 days and 20 days, respectively) (Fig. 3b,c). For subsequent drug formulations, we therefore focused on BB because of its biocompatibility and slow in vivo exchange compared with the other tested solvents.

**Fig. 4:**
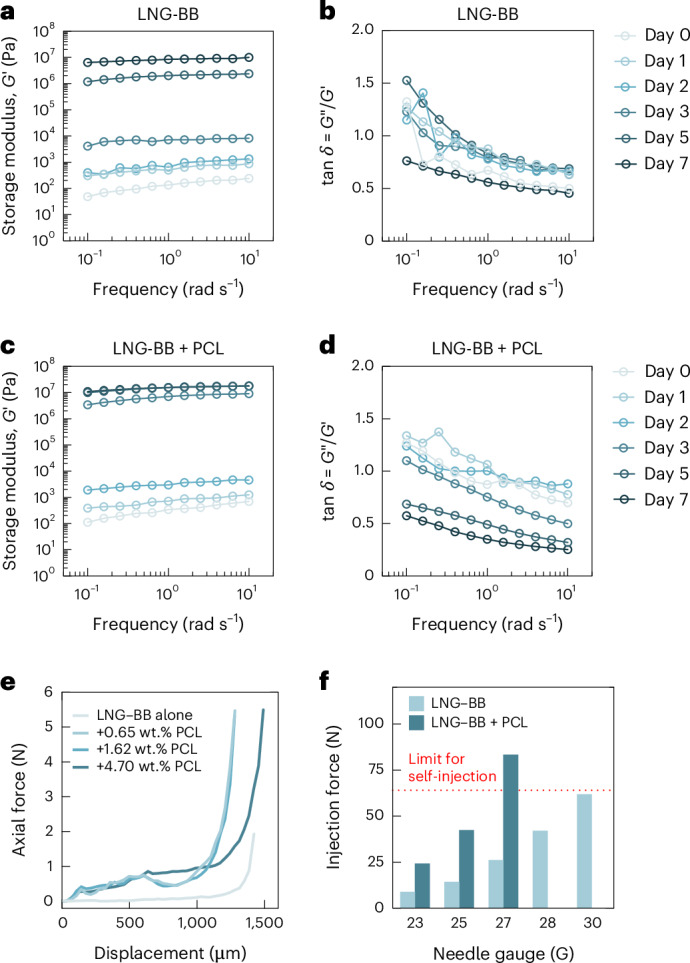
Mechanical and rheological characterization of SLIM. **a**–**d**, Changes in storage modulus (**a**) and tan delta (**b**) of LNG–BB and storage modulus (**c**) and tan delta (**d**) of LNG–BB + PCL in a medium of 5% SDS in PBS over 7 days. **e**, Axial force as a function of compressive displacement on depots self-aggregated from BB formulations. Increasing PCL concentration increases the mechanical integrity of the depots. **f**, Injection force at 6 ml min^−1^ as a function of needle size for LNG–BB (293 mg ml^−1^, light blue) and LNG–BB (293 mg ml^−1^) + PCL (1.62 wt.%, dark blue) formulations, highlighting the self-injectability threshold (red). Source data

In addition to biocompatibility, the mechanical properties are also an important consideration in the design of a long-acting implant^31–33^. Maintaining a monolithic implant over the therapeutic lifetime enables early retrievability and a predictable drug release profile, yet mechanical perturbation in the form of compressive and shear stresses may compromise implant integrity. In rodent studies, such perturbation is introduced during routine handling and enrichment activities. To understand the mechanical properties of LNG–BB depots during the self-aggregation stage, we performed small-amplitude rheological characterization on depots injected in vitro into PBS media comprising 5% SDS. Over 1 week, the storage modulus (*G*′) increased by seven orders of magnitude to a final value of 10 MPa (at an angular frequency (*ω*) = 10 rad s^−1^) as the solvent exchange process proceeded (Fig. 4a). During the depot formation stage, tan delta values were also highly frequency dependent, ranging from low-frequency values above 1, consistent with liquid-like behavior, to values below 1 at higher frequencies, indicating increasingly elastic behavior (Fig. 4b). This is consistent with the depots being composed of discrete microcrystals supported by weak interparticle contacts: at lower frequencies and small strain amplitudes, particles have more time to rearrange in response to shear stress. After 1 week of solidification, however, tan delta was less than 1 for the entire tested frequency range.

To further tune mechanical properties, we investigated the effect of a small (<5 wt.%) amount of PCL on shear and compressive behavior of the depots. As with the LNG–BB formulation alone, formulations comprising 1.6 wt.% PCL solidified dramatically over the course of 1 week (Fig. 4c) to a slightly higher final storage modulus value of 1.8 MPa (at *ω* = 10 rad s^−1^). Notably, tan delta values transitioned to below 1 for the entire tested frequency range after just 3 days, earlier than formulations without polymer (Fig. 4d). Adding PCL also increased the elastic modulus and compressive stress that could be withstood by solidified depots that had been equilibrated for 1 week in exchange media (Fig. 4e and Supplementary Fig. 13). While increasing PCL content did not dramatically affect modulus, depots with more PCL were able to be compressed to higher displacements before fracture. Compared with LNG–BB alone, all depots made with PCL exhibited markedly improved cohesion after compression, suggesting that a small amount of polymer may be important to maintain implant retrievability in the face of exposure to imposed mechanical forces.

Lastly, injectability is a key constraint in formulation optimization. In general, a formulation that can be administered using less than 64 N of force at 6 ml min^−1^ is considered injectable by hand^4^. While previous in situ forming injectable depots mostly rely on 18 G and 19 G needles^6,34^, most self-administrable injectables on the market use smaller needle gauges higher than 23 G (refs. ^16,35,36^). Because our formulation approach does not rely exclusively on a polymer excipient to modulate drug release, we anticipated that our long-acting depots could meet the injectability threshold using much smaller needles than has been reported with similar technologies in the literature owing to the lower concentrations of polymer excipient required. Injection force was measured for the LNG–BB and LNG–BB + 1.62 wt.% PCL formulations using an Instron machine, and the injectability threshold was met through minimum needle sizes of 30 G and 25 G, respectively (Fig. 4f).

### In vivo depot formation and pharmacokinetics

Finally, we performed in vivo pharmacokinetic analysis on female Sprague–Dawley rats to test our hypothesis that our optimized LNG–BB formulations would result in slower LNG release due to reduced depot surface area. Rats were injected with 1 ml of 293 mg ml^−1^ LNG suspensions, and LNG release was evaluated in plasma collected at regular intervals using triple-quadrupole liquid chromatography–tandem mass spectrometry. For an equivalent initial LNG drug dose, a lower average LNG serum concentration corresponds to more remaining LNG within the depot, which, in turn, corresponds to a longer total expected drug release timeframe.

To validate our hypothesis that reduced solvent exchange rates would correspond to slower drug release, we compared our LNG–BB formulations with a control formulation of LNG in PBS. Although NMP also clears from the injection site faster than BB, LNG–NMP was not an appropriate control group to test our hypothesis because LNG is much more soluble in NMP (109.84 ± 21.78 mg ml^−1^) compared with both BB (5.97 ± 2.22 mg ml^−1^) and PBS (insoluble) (Supplementary Fig. 14). Thus, for an equivalent total drug concentration, NMP formulations contain much fewer LNG microcrystals and much more dissolved LNG than BB, making it difficult to interpret whether higher serum concentrations can be attributed to differences in depot morphology. Indeed, we observed that LNG–NMP formulations consistently yielded higher plasma concentrations of LNG than LNG–BB formulations, with a 2.9-fold higher area under the curve (AUC) for 37 days (Supplementary Fig. 15). Notably, the LNG–BB depot also exhibited less and milder inflammation compared with the LNG–NMP depot (Supplementary Fig. 16). Because LNG is even less soluble in PBS than in BB, if higher average serum concentrations were observed for LNG–PBS formulations, then we could be more confident that the differences were attributable to differences in morphology of the aggregated implants.

**Fig. 5:**
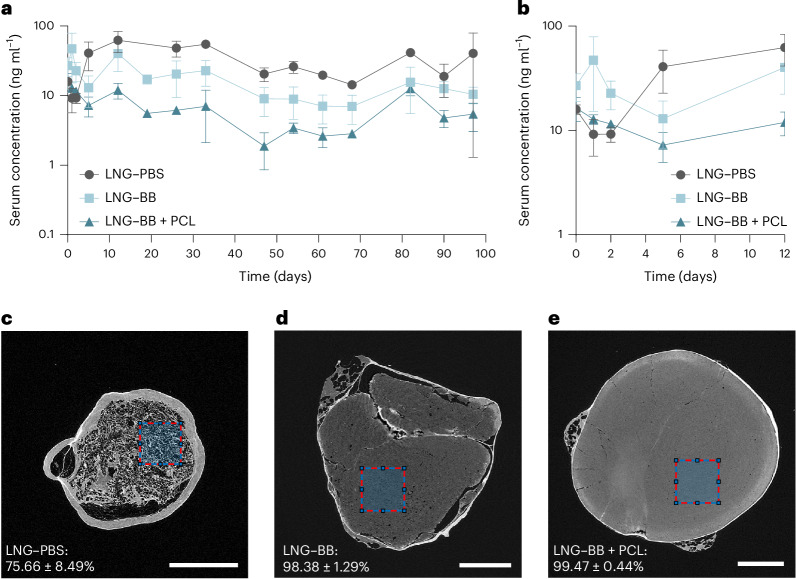
In vivo demonstration of SLIM. **a**, In vivo release of LNG–PBS, LNG–BB and LNG–BB + PCL over 97 days. **b**, Magnified version for the initial time points up to day 12. **c**–**e**, µCT images of LNG–PBS (**c**), LNG–BB (**d**) and LNG–BB + PCL (**e**) depots excised from rats after 97 days, highlighting the higher packing efficiency achieved from formulations with BB compared with PBS. The region of interest (dashed red) is 200 × 200 pixels. Scale bars, 2 mm. *n* = 3 per group. Data represent mean ± s.d. Source data

The release kinetics revealed clear differences in LNG concentrations in plasma over time among the formulations (Fig. 5a and Supplementary Note 2). From day 5 to day 97, the plasma concentration of LNG from the LNG–PBS formulation consistently exceeded that from the LNG–PBS formulations. Specifically, the AUC for the PBS formulation was quantified at 3,401 ± 262.7 ng ml^−1^ per day, approximately 2.2-fold higher than the AUC observed for the LNG–BB formulation, which was 1,537 ± 163.2 ng ml^−1^ per day. With the addition of PCL, serum LNG concentrations throughout the same time period were reduced even further: The AUC for the LNG–BB + PCL formulation was 605.3 ± 49.98 ng ml^−1^ per day, demonstrating a 2.5-fold reduction compared with the LNG–BB formulation and a 5.6-fold reduction compared with the LNG–PBS formulation. Interestingly, during the first 5 days, the incorporation of PCL attenuated the initial LNG release rate, whereas the LNG–BB-only formulation showed higher serum concentrations than the LNG–PBS formulation (Fig. 5b). These results highlight the complex interplay between solvent choice and polymer addition in controlling the release kinetics of a hydrophobic drug such as LNG. The findings provide insights into formulation strategies that could potentially optimize therapeutic efficacy in drug delivery systems.

We hypothesize that the promising long-term in vivo release kinetics of the LNG–BB formulations can be attributed to a two-stage process of depot formation, both of which are favorable for sustained release of LNG: a first stage during depot solidification that, based on solvent clearance data (Fig. 3c), may be around 1 month, and a second stage post-solidification wherein the depot is fully dense and there is no remaining solvent. Drug release during the first phase could be a function of LNG partitioning into the BB phase, and further partitioning from the BB to the surrounding interstitial fluid. Meanwhile, in the second stage, the favorable release kinetics can be attributed to reduced porosity in the aggregated implant due to the slower exchange of BB with its surroundings compared with PBS. Reduced porosity corresponds to reduced water infiltration, which enables slower and controlled surface erosion of a smaller surface area compared with the LNG–PBS control. To validate this hypothesis, microcomputed tomography (µCT) was used to achieve high-resolution imaging and 3D reconstruction of the depots retrieved from the animal study after euthanasia at 97 days. As expected, higher packing efficiency was achieved for the depots aggregated from formulations of slow-exchanging solvents (98.4 ± 1.3% for LNG–BB and 99.5 ± 0.4% for LNG–BB + PCL) compared with the LNG–PBS control (75.7 ± 8.5%) (Fig. 5c–e). The µCT analysis therefore corroborates our proposed mechanism to extend drug release from injectable implants by using slow-exchanging solvents to improve drug microcrystal compaction.

Two competing factors influence the relative dominance between these two stages by affecting the LNG concentration in the saturated suspension depot in opposing ways: (1) diffusion of solvent (BB) and (2) diffusion of solute (LNG) away from the injection site (Supplementary Fig. 17). As BB diffuses out from the depot, it promotes the precipitation of drug crystals by increasing LNG concentration above its saturation point (*C*_sat_). This solvent diffusion depends primarily on the miscibility between BB and the aqueous medium. Meanwhile, LNG can also diffuse across the BB–body fluid interface in a process called liquid–liquid extraction. The solute transfer across the interface is governed by the partition coefficient, which depends on the difference in drug solubility within the two solvent phases. LNG continually transfers out of the BB phase, promoting further dissolution of the suspended LNG microcrystals. The rate of this transfer is governed by the concentration gradient across the interface and the thickness of the diffusion layers within both phases.

If solvent diffusion, which induces precipitation, is faster than solute transfer, which encourages further dissolution, the second stage of drug release becomes more dominant. Conversely, if solute diffusion prevails, a substantial amount of LNG crystals may dissolve before compaction occurs. In this latter case, it is likely that only the first stage would be observed. Our LNG–BB formulation strikes a balance in which solvent diffusion is fast enough to observe the second stage of release, while the solute diffusion is still slow enough to enable the microcrystals to remain saturated and form into a highly compacted dense structure.

## Discussion

SLIM is a design for long-acting injectable implants based on the in situ self-aggregation of drug crystals driven by the slow exchange of BB with the surroundings at the injection site. Using LNG as a proof-of-concept demonstration to show the potential of this technology, our results suggest that the rate of solvent exchange profoundly influences the structural integrity and drug release dynamics of the self-aggregated depot, with slow-exchanging BB resulting in slower drug release rates and, therefore, longer total release timeframes. The solvent-exchange-mediated aggregation mechanism allows injection through needles as small as 30 G (with no polymer) and 25 G (with a low amount of polymer excipient) while extending the drug release timeframe relative to aqueous suspensions by promoting structural integrity and minimizing surface area via microcrystal compaction. In vivo evaluations validated these theoretical advantages, confirming that the compacted microcrystal structure of SLIM greatly reduces average drug release rate. In addition, histological analysis confirmed that the components used in SLIM were well tolerated, which supports its strong safety profile and potential for broader clinical applications. Specifically, in applications such as contraception, SLIM’s capability for prolonged drug release could dramatically reduce the frequency of administration compared with current self-administrable options such as Depo-Provera and Sayana Press, potentially transforming the landscape of contraceptive technology.

Future work will investigate the long-term in vivo pharmacokinetics of self-aggregated implants to elucidate the relationship between dose, duration and injectability. Notably, because improvements in drug release kinetics could be observed simply by improving compaction of drug microcrystals, we anticipate that a major advantage of the SLIM system will be the ability to increase drug loading by increasing microcrystal concentration without requiring more polymer. Because the viscosity of solid suspensions tends to increase much more slowly as a function of loading compared with polymeric solutions (Supplementary Fig. 18)^37^, we expect that this characteristic will enable us to boost dosages without substantially compromising the ability to inject formulations through small needles compatible with self-administration.

Furthermore, it is likely that depot properties depend on the biochemical and mechanical properties of subcutaneous tissue at the injection site. As such, understanding how drug release profiles differ between injection locations and animal models is important to predict how formulations will perform in humans. Moreover, while we have established that mechanical properties such as modulus are tunable, a clear definition of the mechanical forces that an implant must withstand in vivo to maintain long-term integrity and enable early retrieval is needed. Although this work focused on the contraceptive drug LNG, we anticipate that our design approach will be applicable to other hydrophobic drugs as well, which is important because the majority of new drugs developed in the pharmaceutical industry are poorly soluble in physiological fluids^38^. While LNG is relatively potent, our formulation approach is well suited to less-potent drugs as well, as they are even more dependent on high loading to achieve long-term release. To expand the platform potential of in-situ*-*aggregated compacted microcrystal implants, future work will investigate how physicochemical properties of different drugs affect the self-aggregation and depot characteristics observed for LNG.

## Methods

### Materials and formulations

LNG was supplied by Austin Chemical (CAS no. 797-63-7). BB (CAS no. 120-51-4), BA (CAS no. 100-21-6) and NMP (CAS no. 872-50-4) were purchased from Sigma-Aldrich. PCL (molecular weight 50 kDa) was purchased from Polyscience. In vitro exchange media comprised PBS (Thermo Fisher Scientific, pH 7.4) with varying amounts of SDS (Thermo Fisher Scientific, CAS no. 151-21-3). All LNG formulations were prepared by using a FlackTek Speedmixer to homogenize LNG powder with either solvent alone or a polymer solution comprising predissolved polymer in solvent. Formulations were allowed to equilibrate for at least 1 day before the solvent reached the saturated concentration of LNG, and all formulations were vortexed for 15 s immediately before use. LNG depots for in vitro timelapse imaging and mechanical testing were formed in glassware using a ratio of 10 µl formulation to 5 ml exchange medium.

### In vitro characterization

Macroscale in vitro timelapse images were obtained using a Canon EOS R5 digital camera equipped with a Canon RF 24-105 mm F4-7.1 objective lens. A light ring was placed over the vials to minimize the light interference effects due to the multiple light sources present in the laboratory. Contact angle measurements were carried out via ImageJ Contact_Angle.jar plugin using the ellipse best-fit, as the one leading to the most precise fitting method for the droplets under analysis. The procedure described by the plugin author was followed. The contact radius was measured in ImageJ by calibrating every frame with the respective scale bar and measuring the segment of droplet that was in contact with the substrate.

LNG microcrystal dissolution was imaged using a Leica DMi1 optical microscope equipped with a FLEXACAM C1 camera. Samples were imaged in either one- or eight-chambered coverglass with a coverslip 1.5H grade (thickness of 0.170 ± 0.005 mm), and the microscope was calibrated in-house using a microruler. Brightfield microscopy was used to obtain videos of LNG crystal growth, using a Zeiss AxioObserver Z1 stand and camera (Zeiss Axiocam 305 mono camera). Videos were acquired with 0.028-s time steps using a 63× objective with a numerical aperture of 1.4 with oil immersion. Scanning electron microscopy was performed using a Hitachi FlexSEM TM-1000 II in high-vacuum mode with an accelerating voltage of 3 kV. Before imaging, samples were dried for at least 24 h at room temperature and gold-coated by sputtering for 2 min with a JEOL USA SmartCoater. Particle size analysis was performed using Mountain Maps software, version 8.2.

Solubility values were obtained by preparing supersaturated suspensions of LNG in solvent, equilibrating the suspensions for 24 h, extracting the supernatants after centrifugation and then obtaining the LNG concentration in the supernatant using high-performance liquid chromatography. Reported solubility values are averaged over five samples per solvent. Powder XRD of all LNG samples was measured using the ANalytical’ X’Pert PRO XRPD, with a 1.8 kW sealed X-ray tube source, using a Cu target, and a vertical circle goniometer. All LNG samples were measured in a Si zero background plate with a cavity 18 mm × 0.2 mm deep.

Injection force was measured using an Instron 5943 Series Universal Testing System with a 500 N load cell. A customized stage was 3D printed to support the syringe during the injection study. All measurements used standard 1-ml Luer lock syringes with a compression rate of 5.72 mm s^−1^, which is equivalent to 6 ml min^−1^. Compression testing and shear rheometry were both performed using a Discovery Hybrid Rheometer 3 from TA Instruments with an 8-mm cross-hatched parallel plate. Depots were compressed at a rate of 35 µm s^−1^ while submerged in 10 ml of PBS using the rheometer’s solvent trap. Oscillatory shear rheology measurements were obtained using a strain value of 0.1% and 25 °C, with a frequency sweep range of 0.1–100 rad s^−1^ and 500 µm gap size.

### Initial toxicity assessment of solvent carriers

All animal testing was approved by the Committee on Animal Care at the Massachusetts Institute of Technology. Rodent studies were performed using female Sprague–Dawley rats, and subcutaneous injections were administered for all experiments.To assess the toxicity, BB, BA and PBS were injected subcutaneously into rats at doses of 240 μl kg^−1^ (low dose) and 1,600 μl kg^−1^ (high dose). The rats were monitored for signs of acute and chronic toxicity, such as weight loss. On days 3 and 28 post-injection, the rats were euthanized. The tissue from the injection site, as well as tissues from the liver, kidney, spleen and heart, were collected for histological and multiorgan analysis to assess systemic effects. These tissues were immersed in 10% neutral buffered formalin for 48 h, then transferred to 70% ethanol and stored at room temperature until further processing. Each tissue sample was subsequently prepared into slides, stained with hematoxylin and eosin, and analyzed using an optical microscope.

### In vivo ultrasound imaging

A 34-day ultrasound imaging study was conducted to assess the clearance of the solvents used in this research. A total of 0.5 ml each of BB, NMP and PBS was injected subcutaneously into a single Sprague–Dawley rat under anesthesia (*n* = 1 per group). Ultrasound and photoacoustic imaging were performed using the Vevo LAZR-X Photoacoustic and Micro-Ultrasound Imaging System (FUJIFILM VisualSonics) with an MX250S transducer at a frequency of 21 MHz. The transducer was moved across the skin over the subcutaneous injection site using a 3D stepper motor to obtain a comprehensive 3D dataset. Images were processed and rendered using VevoLAB 3.2.0 software (FUJIFILM VisualSonics).

### In vivo pharmacokinetic studies

A 97-day in vivo study was conducted to assess the pharmacokinetics of LNG–PBS, LNG–BB and LNG–BB + PCL formulations in Sprague–Dawley rats. In addition, another 37-day in vivo study evaluated LNG–BB, LNG–NMP and LNG–NMP + PCL formulations. All experiments involving rats were conducted in accordance with a protocol approved by the Committee on Animal Care at the Massachusetts Institute of Technology. Rats were subcutaneously injected with 0.5 ml of the formulations using a 25 G needle. Blood was collected from the tail vein at each time point into serum separator tubes. All samples were stored at −80 °C until pharmacokinetic analysis was performed after centrifugation. The serum concentration of LNG was measured by triple-quadrupole liquid chromatography–tandem mass spectrometry.

### Histopathological analysis

After the completion of the pharmacokinetic evaluation and the euthanasia of rats in the 37-day study with LNG–BB, LNG–NMP and LNG–NMP + PCL formulations, the targeted depot and adjacent tissues, including the underlying subcutaneous fat, were excised for histological analysis. Initially, the specimens were immersed in 10% neutral buffered formalin for 48 h, then transferred to 70% ethanol, which was maintained at ambient temperature pending further processing. The preparation of the fixed skin specimens involved making a central longitudinal section to ensure the depot was centrally aligned, before embedding the specimens in paraffin blocks. Sections with a thickness of 5 µm were then prepared from these embedded samples and subjected to staining with hematoxylin and eosin as well as Masson’s trichrome. Sections were assessed for the type and severity of inflammation, the extent of fibrosis and the presence of necrosis by a board-certified veterinary pathologist.

### Ex vivo characterization

µCT was performed using a Nikon X-tek HMXST 225. Before characterization, the ex vivo depots were prepared directly within the tissue by freezing in liquid nitrogen immediately upon explantation and lyophilizing with a decreasing temperature ramp rate of 1 °C min^−1^. They were then left in the vacuum chamber for 3 days, at a maximum shelf temperature of −40 °C and 0.1 mbar. The samples were then stored in a −20 °C freezer until they were brought to the µCT where they were glued with commercially available epoxy to a needle-sample holder. A molybdenum target was used to generate the X-ray beam. The voltage and beam current were set to 45 kV and approximately 220, respectively. To maintain a consistent pixel resolution, the magnification value was set to approximately 29.1 across all samples. Data were analyzed with a self-developed MATLAB code to combine images and calculate local porosity in the analyzed samples. Each sample was first analyzed by combining all the images in a GIF file for quick qualitative assessment of the overall morphology of the depot. Subsequently, three representative slices (*n* = 3) were selected for each sample. The average porosity and s.d. were computed by applying a pixel-intensity threshold to distinguish which pixels were LNG and which were empty spaces in the depot, thus allowing the assessment of the packing efficiency. Results were then presented as *μ* ± *σ*, where *μ* and *σ* are respectively the arithmetic mean and the s.d. of the estimated porosity value.

### Statistical analysis

All quantitative measurements were performed with at least three independent replicates. The data are presented as mean ± s.d.

### Reporting summary

Further information on research design is available in the Nature Portfolio Reporting Summary linked to this article.

## Supplementary information

Supplementary Information Supplementary Figs. 1–18, Notes 1 and 2 and Tables 1 and 2.

Reporting Summary

Supplementary Table 1 Review of long-acting injectable contraceptives.

Supplementary Table 2 Review of long-acting injectables for various active pharmaceutical ingredients.

Supplementary Data 1 μCT images.

## Source data

Source Data Fig. 2 Image analysis of depot compaction.

Source Data Fig. 4 Rheology and mechanical test (compression, injection force).

Source Data Fig. 5 Pharmacokinetics data.

## Data Availability

All the data supporting the results in this study are available within the Article and its Supplementary Information. Source data are provided with this paper. Additional data may be requested from the authors.

## Code availability

MATLAB scripts used for the µCT analyses are available via GitHub at https://github.com/sangpark-mit/slim-microCT-NatChemE.git.
