# Supplementary material for: Self-aggregating long-acting injectable microcrystals
Source: Nat Chem Eng. 2025 Mar 24;2(3):209–19. doi: 10.1038/s44286-025-00194-x (PMC11932925; doi:10.1038/s44286-025-00194-x)
Supplement: Supplementary file 1 — Supplementary Figs. 1–18, Notes 1 and 2 and Tables 1 and 2. [file 44286_2025_194_MOESM1_ESM.pdf]

nature chemical engineering

Supplementary information https://doi.org/10.1038/s44286-025-00194-x

Self-aggregating long-acting injectable microcrystals

In the format provided by the authors and unedited

Table of Contents

Supplementary Table 1 ... 3
Supplementary Table 2 ... 5
Supplementary Figure 1 ... 7
Supplementary Figure 2 ... 8
Supplementary Note 1 ... 9
Supplementary Figure 3 ... 11
Supplementary Figure 4 ... 12
Supplementary Figure 5 ... 13
Supplementary Figure 6 ... 14
Supplementary Figure 7 ... 15
Supplementary Figure 8 ... 16
Supplementary Figure 9 ... 17
Supplementary Figure 10 ... 18
Supplementary Figure 11 ... 19
Supplementary Figure 12 ... 20
Supplementary Figure 13 ... 21
Supplementary Figure 14 ... 22
Supplementary Figure 15 ... 23
Supplementary Figure 16 ... 24
Supplementary Note 2 ... 25
Supplementary Figure 17 ... 26
Supplementary Figure 18 ... 27
References ... 28

Supplementary Table 1, 2 | Comparative Analysis of Self-assembling Long-acting Injectable Microcrystals (SLIM) and Existing Long-acting Injectable Technologies: Focus on Microparticle Suspensions and In Situ Forming Implants

We conducted a literature review to compare the highlighted aspects of Self-assembling Long-acting Injectable Microcrystals (SLIM) with other existing works in long-acting injectable (LAI) technologies, mostly focusing on microparticle suspensions and in situ forming implants (ISFIs), as well as relevant commercialized products. ISFIs encompass various types of in situ forming implants, including systems based on cross-linking or thermosensitive hydrogels, while "ISFI (phase inversion)" are most relevant for comparison to our system. Table 1 covers LAIs for contraception throughout all time periods and includes both intramuscular and subcutaneous injectables due to the limited number of products. Table 2 focuses solely on subcutaneous injectables and covers journal papers on LAIs for other APIs over the past 5 years, as well as commercialized products from the past 10 years.

For both charts, the 'Polymer:Drug ratio' is calculated by normalizing the weight ratio between the drug and polymer to a drug value of 1. The 'Duration of release' is based on the longest time points reported from in vivo studies in journal papers and the intended use for commercial products. 'Needle size' is derived from the gauge of the needle used in the included in vivo studies and the needle gauge of the products. 'Drug concentration' represents the concentration of drug per volume for in situ forming implants, which is not applicable (N/A) for microparticle suspensions. 'Polymer/solvent or dispersion medium' specifies the polymer and solvent used for in situ forming implants, or the polymer and suspension medium used for injection in microparticle types. For journal papers, when multiple formulations are presented, we select the lead formulation that was chosen for the final in vivo study. We also focus on the formulation that exhibits the lowest polymer-to-drug ratio, aligning with the purpose of this literature review.

Supplementary Table 1. Review of Long-acting Injectable Contraceptives

Source	Authors	Date	Type	Polymer/Drug ratio (wt/wt)	Duration of release	Needle size	Drug concentration (mg/ml)	Polymer portion (wt%)	Polymer/Solvent or dispersion medium	Indications	API	Ref.
This work		2024	SLIM	0.0625	> 97 days	25 G	293	1.6	Polymer: PCL (50k) Solvent: Benzyl benzoate	Contraception	Levonorgestrel (LNG)	
This work		2024	SLIM	0	> 97 days	25 G* (injectability <64 N through 28 G needle was confirmed in vitro)	293	0	Polymer: None Solvent: Benzyl benzoate	Contraception	Levonorgestrel (LNG)	
Journal paper	Young et al.	2023	ISFI (phase inversion)	0.35	90 days	19 G	535.8	11.17318436	Polymer: PLGA (50:50, 10k) Solvent: 1:1 NMP/DMSO	Prevention of HIV and pregnancy	Cabotegravir/Etonogestrel	[1]
Journal paper	Tang et al.	2022	Microparticle suspension	0.3333	98 days	Not specified	N/A	25	Polymer: PLA Dispersion medium: sterile PBS with 3% (w/v) carboxymethyl cellulose	Contraception	Etonogestrel	[2]
Journal paper	Janagam et al.	2017	ISFI (phase inversion)	3.92	7 months	21-23 G	85	23.5 (PLA: 18.8%, PLGA 50:50: 4.7%)	Polymer: PLGA (50:50), PLA Solvent: NMP/TEC (triethyl citrate)	Contraception	LNG	[3]
Journal paper	Zhang et al.	2013	ISFI (phase inversion)	1.05	3 months	21 G	Not specified	16.8	Polymer: 80:20 P(CL/DL-LA) Solvent: NMP	Contraception (male)	Testosterone undecanoate	[4]
Journal paper	Puthli and Vavia	2009	Microparticle suspension	5	28 days	22 G (intramuscular)	N/A	83.3	Polymer: PLGA Dispersion medum: saline	Contraception	LNG	[5]
Journal paper	Sun et al.	2008	Microparticle suspension	0.43	35 days	Not specified	N/A	30.1	Polymer: PLGA Dispersion medum: 0.5% CMC-Na and 0.05% Tween-80 solution	Contraception	Gestodene and ethinyl estradiol	[6]
Journal paper	Wang et al.	2005	Microparticle suspension	3.78	168 days	23 G (intramuscular)	N/A	79.09	Polymer: PLGA Dispersion medium: 0.9% saline solution containing 2% (w/w) sodium carboxymethylcellulose (CMC–Na) and 1% (w/w) Tween 20	Contraception	LNG	[7]
Journal paper	Dhanaraju et al.	2006	Microparticle suspension	5	15 weeks	18 G (intramuscular)	N/A	83.3	Polymer: PLGA Dispersion medium: physiological saline containing 0.1% Tween-80	Contraception	Ethinyl estradiol and LNG	[8]

FDA approved product /Under clinical trial	Manufacturer	Year of Approval	Type	Polymer/Drug ratio (wt/wt)	Duration of release	Needle size	Drug concentration (mg/ml)	Polymer portion (wt%)	Polymer/Solvent or dispersion medium	Indications	API	Ref.
Sayana press	Pfizer	2021	Particulate suspension	N/A	3 months	23 G	160	N/A	Polymer: N/A<br>Dispersion medium: PEG 3350 aqueous solution	Prevention of pregnancy	Medroxyprogesterone acetate	9
Depo-provera 104	Pfizer	2016	Particulate suspension	N/A	3 months	26 G	160	N/A	Polymer: N/A<br>Dispersion medium: PEG 3350 aqueous solution	Prevention of pregnancy	Medroxyprogesterone acetate	10
Depo-provera CI	Pfizer	2010	Particulate suspension	N/A	4 months	22 G	150	N/A	Polymer: N/A<br>Dispersion medium: PEG 3350 aqueous solution	Prevention of pregnancy	Medroxyprogesterone acetate	11

Supplementary Table 2. Review of Long-acting Injectables for Various APIs

Source	Authors	Date	Type	Polymer/Drug ratio (wt/wt)	Duration of release	Needle size	Drug concentration (mg/ml)	Polymer portion (wt%)	Polymer/Solvent or dispersion medium	Indications	API	Ref.
This work		2024	SLIM	0.0625	> 97 days	25 G	293	1.6	Polymer: PCL (50k) Solvent: Benzyl benzoate	Contraception	Levonorgestrel (LNG)	
This work		2024	SLIM	0	> 97 days	25 G* (injectability <64 N through 28 G needle was confirmed in vitro)	293	1.6	Polymer: None Solvent: Benzyl benzoate	Contraception	Levonorgestrel (LNG)	
Journal paper	Nakhla et al.	2024	Microparticle suspension	10	28 days	Not specified	3.33	N/A	Polymer: PLGA Dispersion medium: 1 mL of 1x Dulbecco's phosphate-buffered saline (DPBS)	Cystic fibrosis treatment	Ivacaftor	12
Journal paper	Young et al.	2023	ISFI (phase inversion)	0.286	6-11 months	16-18 G	500	11.7	Polymer: PLGA (50:50, 10k) Solvent: NMP: DMSO (1:1, w/w)	HIV prophylaxis	Cabotegravir	13
Journal paper	Wang et al.	2023	Microparticle suspension	9	42 days	19 G	N/A	90	Dispersion medium: aqeuous solution with 0.5% low viscosity carboxymethyl cellulose (CMC), 0.1% w/v Tween 80, and 5% d-mannitol	Syndromic obesity treatment	Setmelanotide	14
Journal paper	Zhao et al.	2023	ISFI	12	7 days	20 G	Not specified	55.8	Polymer: PLA Solvent: NMP (1:07 ratio)	Parkinson's Disease	Resagiline	15
Journal paper	Rajadhyaksha et al.	2023	ISFI	25.21	14 days	23 G	Not specified	99.95	Polymer: chitosan, B-glycerophosphate, Hyaluronic acid	Broad chronic disease in need of protein therapeutics	Trastuzumab	16
Journal paper	Wu et al.	2023	ISFI	16.62	20 days	20 G	Not specified	85.4	Polymer: soya phosphatidyl choline (SPC), glycoerol dioleate (GDO) Solvent: ethanol	Parkinson's Disease	Rotigotine	17
Journal paper	Kim et al.	2022	Microparticle suspension	5	1 and 3 months	Not specified	N/A	56.25	Polymer: PLA Dispersion medium: diluent, consisting of 0.5% sodium carboxymethylcellulose, 5% D-mannitol, 0.1% polysorbate 80, and water	Hormonal disorders treatment	Leuprolide acetate	18
Journal paper	Kim et al.	2022	ISFI	2.08	4 months	19 G	202	14.1	Polymer: PLGA Solvent: DMSO, NMP	Tuberculosis treatment	Rifabutin	19
Journal paper	Choi et al.	2021	Microparticle suspension	9	1 month	Not specified	63.84	15	Polymer: PLGA 7525A Dispersion medium: .5% sodium	Alzheimer's disease inhibition	Donepezil	20

									carboxymethylcellulose, 5% mannitol, 0.1% polysorbate 80, and water			
Journal paper	Zhai et al.	2020	Microparticle suspension	15	1 month	Not specified	6.78	93.75	Polymer: PLGA (50:50)	Treatment for type 2 diabetes	Glucagon-like peptide-1 receptor agonist exenatide	21
Journal paper	Benhabbour et al.	2019	ISFI (phase inversion)	1.23	350 days	19 G	300	26.2	Polymer: PLGA (50:50, 27k) Solvent: NMP	HIV treatment/prophylaxis	MK-2048	22

**FDA approved product /Under clinical trial**	**Manufacturer**	**Year of Approval**	**Type**	**Polymer/Drug ratio (wt/wt)**	**Duration of release**	**Needle size**	**Drug concentration (mg/ml)**	**Polymer portion (wt%)**	**Polymer/Solvent or dispersion medium**	**Indications**	**API**	
Brixadi	Braeburn Inc	2023	ISFI	Not specified	1 month	23 G	356 mg/mL (monthly)	Not specified	Not specified	Opioid use treatment	Buprenorphine	23
Sublocade	Curia Global	2021	ISFI	1.78	1 month	19 G	200 mg/mL (highest dose)	32	Polymer: PLGA Solvent: NMP	Substance Use Disorder	Buprenorphine	24
Camcevi	Foresee Pharmaceuticals	2021	Microparticle suspension	4.38	6 months	18 G	Not specified	50	Polymer: PLA Solvent: NMP	Prostatic Cancer	Leuprolide	25
Fensolvi	Tolmar	2020	ISFI	3.67	6 months	18 G	120 mg/mL	44	Polymer: PLG	Precocious Puberty	Leuprolide acetate	26
Bydureon	AstraZeneca	2018	Microparticle suspension	18.6	7 days	23 G	3.08 mg/mL	93	Polymer: PLGA	Type 2 Diabetes mellitus	Exenatide	27
Perseris	Indivior	2018	ISFI	2.53	1 month	18 G	150 mg/mL (highest dose)	38	Polymer: PLGH, Solvent: NMP	Schizophrenia	Risperidone	28
Eligard	Tolmar	2016	ISFI	3.67	6 months	18 G	120 mg/mL (highest dose)	44	Polymer: PLG Solvent: NMP	Prostatic cancer	Leuprolide acetate	29
Norditropin	Biopartners and LG Life Sciences	2016	Microparticle suspension	Not specified	1 week	26 G	10 mg/mL (highest dose)	Not specified	Not specified	GH Deficiency, Turner Syndrome	Somatropin	30
Sustol	Heron Therapeutics	2016	ISFI	49	1 week	18 G	25 mg/mL	98	Polymer: tri(ethylene glycol) poly (orthoester) (TEG-POE) and polyethylene glycol monomethyl ether	Vomiting from Chemotherapy	Granisetron	31
Nutropin AQ	Genentech	2014	Microparticle suspension	Not specified	4-5 weeks	29G, 30G, 31 G all compatible	10 mg/mL (highest dose)	Not specified	Not specified	GH Deficiency	Somatropin	32

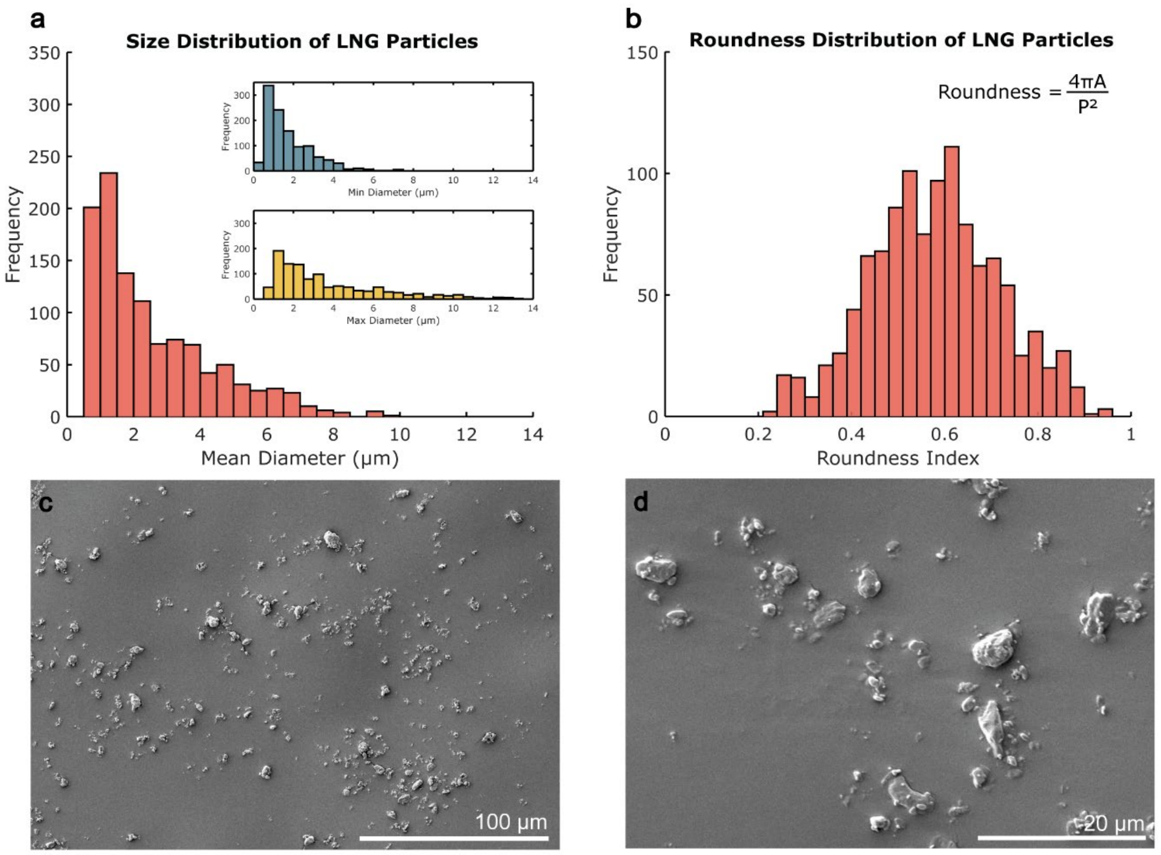

Supplementary Figure 1: a-b LNG microcrystal characterization. (a) LNG particle size distribution as represented by the mean, minimum, and maximum diameter distribution and (b) roundness distribution. The roundness index is given by four times pi times the area divided by the perimeter squared. A roundness index of 1.0 indicates a perfectly spherical particle and decreasing values are representative of increasingly elongated and asymmetrical particles. Analysis was conducted with 1121 particles. c-d Scanning electron microscopy images of LNG powder at (c) 500 and (d) 2000 times magnification.

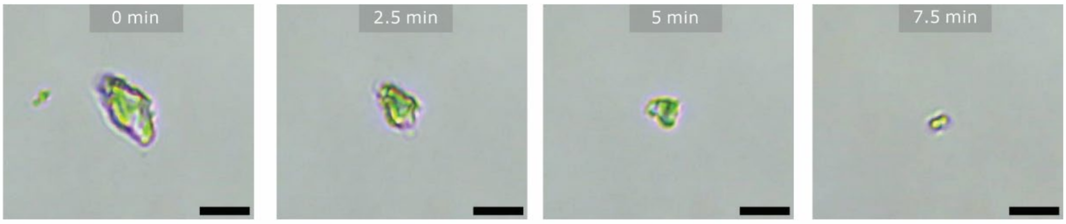

Supplementary Figure 2: Timelapse of LNG microcrystal dissolution in PBS + 10% SDS medium for 0, 2.5, 5 and 7 minutes time interval imaged using Leica DMi1 optical microscope equipped with a FLEXACAM C1 camera and coverslip-bottomed wells. Scale bar is 3 µm.

Supplementary Note 1 | Effect of Microcrystals Packing on External Surface Area (Derivation)
We hypothesize that, if efficiently packed together, jammed hydrophobic drug crystals could enable long-term, injectable, and retrievable implants. This hypothesis relies on the fact that LNG microcrystals are water-impermeable solids and LNG molecules are released into the surrounding media via surface erosion. Therefore, assuming no water infiltration, by efficiently compacting the microcrystals together, it is theoretically possible to minimize the external surface area of the assembled depot that is interfacing with the surrounding environment and thus slow down release. Consequently, higher packing efficiency results in smaller depot volume, external surface area and thus release for the same amount of microcrystals. Furthermore, more efficient compaction of LNG microcrystals is expected to further minimize water infiltration into the bulk of the implant thus improving its mechanical properties and leading to steady dissolution by surface erosion over time. To assess the implications of this hypothesis, a simple analytical model was developed to estimate the impact of packing efficiency on the external surface area exposed to the outer medium, and thus release rate, using characteristic parameters from the system under study. The packing efficiency or packing fraction (φ) of a granular system, i, is defined as the ratio of the volume occupied by the system (Vi) over the volume of the overall space available to be occupied and is mathematically expressed as:

$$\varphi = \frac{V_i}{V_{Space}} \quad (1)$$

It is possible to estimate the effect of packing efficiency by comparing the surface area of a dilute suspension of n LNG microcrystals against the external surface area of the same number of microcrystals but jammed together with a packing efficiency φ. Assuming perfectly spherical and monodispersed microcrystals, it is possible to equate the volume occupied by the microcrystals in the two different morphological configurations, dilute microcrystals suspension (Suspension) and compacted depot (Depot), and solve to estimate the compacted depot diameter, as described by the following equation:

$$\overbrace{n\frac{\pi d^3}{6}}^{V_{Suspension}} = \overbrace{\varphi\frac{\pi d'^3}{6}}^{V_{Depot}} \rightarrow d' = d\left(\frac{n}{\varphi}\right)^{\frac{1}{3}} \quad (2)$$

Where d and d′ are the diameter of the single LNG microcrystal and of the compacted depot respectively. Now it is possible to estimate the external surface areas in the two cases under analysis:

$$A_{Suspension} = n\pi d^2 \quad (3)$$

$$A_{Depot} = \pi d'^2 = \overbrace{\pi d^2}^{\frac{A_{Suspension}}{n}} \left(\frac{n}{\varphi}\right)^{\frac{2}{3}} \quad (4)$$

The suspension equation assumes that all the microcrystals are dispersed in space and are far away enough from each other in order to not affect the mutual release profiles while the depot equation assumes that the compacted depot has perfectly spherical shape and neglects the effect of the roughness represented by the microparticles on the outer surface, which would lead in practice to a non-smooth interface with the surrounding environment. To estimate the effect of compaction on the external surface area, a back-of-the-envelope calculation was developed using parameters from the systems under analysis. Assuming an injection volume (V_{Inj}) of 1mL, a LNG solid volume fraction (ϕ_{LNG}) of 0. 3 and an average diameter of LNG drug crystals (d) of 5 μm it is possible to estimate the approximate number of crystals per injection:

$$V_{\text{Tot Drug}} = n\frac{\pi d^3}{6} = \phi_{LNG}V_{Inj} \quad \longrightarrow \quad n = \frac{6\phi_{LNG}V_{Inj}}{\pi d^3} \approx 4.6 \cdot 10^9 \tag{5}$$

Inserting the above equation into the surface area equations for suspension and depot, and assuming random close packing (RCP) as the packing efficiency, results in an estimated 1200-fold reduction in the external surface area of the compacted depot compared to the diluted suspension. This indicates that by simply compacting together the same amount of drug crystals can theoretically achieve a significantly slower release rate.

$$\frac{A_{\text{Suspension}}}{A_{\text{Depot}}} \approx 1200 \tag{6}$$

The higher the packing efficiency is, the smaller is the external surface area available to surface erosion suggesting that the two are theoretically related by a power law $A \propto \varphi^{-2/3}$. From basic transport phenomena relations, it is possible to recognize that the release rate of a dissolving solid volume is directly proportional to the external surface area. This leads to the same relation of the release rate as a function of packing efficiency:

$$\text{Depot}_{\text{Release Rate}} \propto A_{\text{Depot}} \propto \varphi^{-\frac{2}{3}} \tag{7}$$

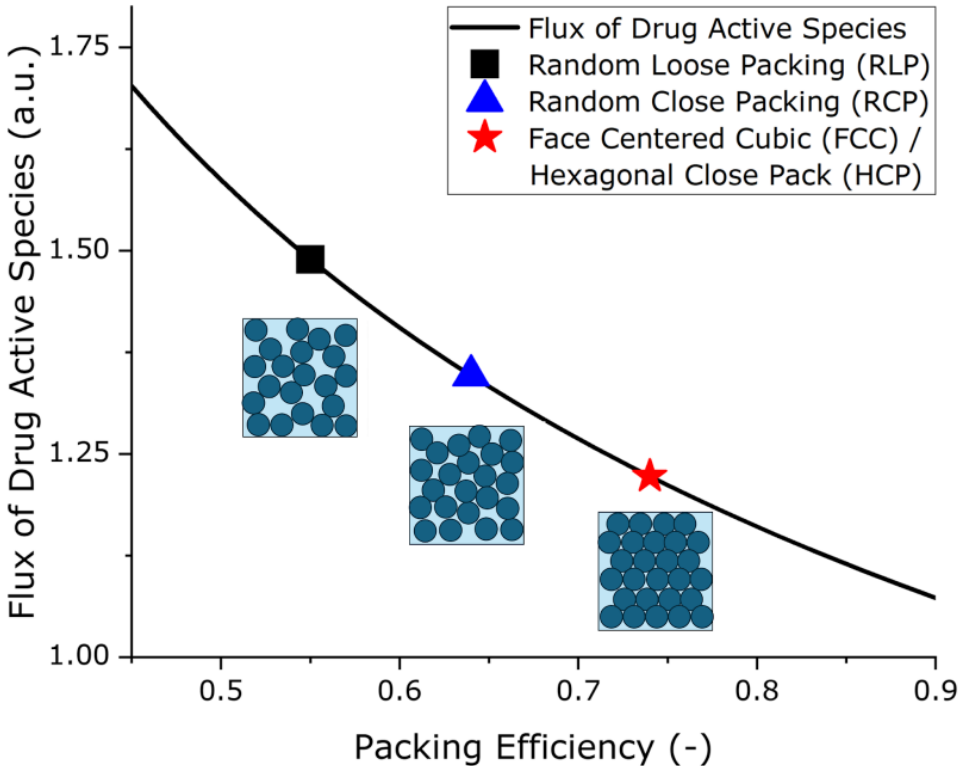

Supplementary Figure 3: Dependency of drug release rate as a function of packing efficiency of the compacted depot, as derived in Supplementary Discussion 1.

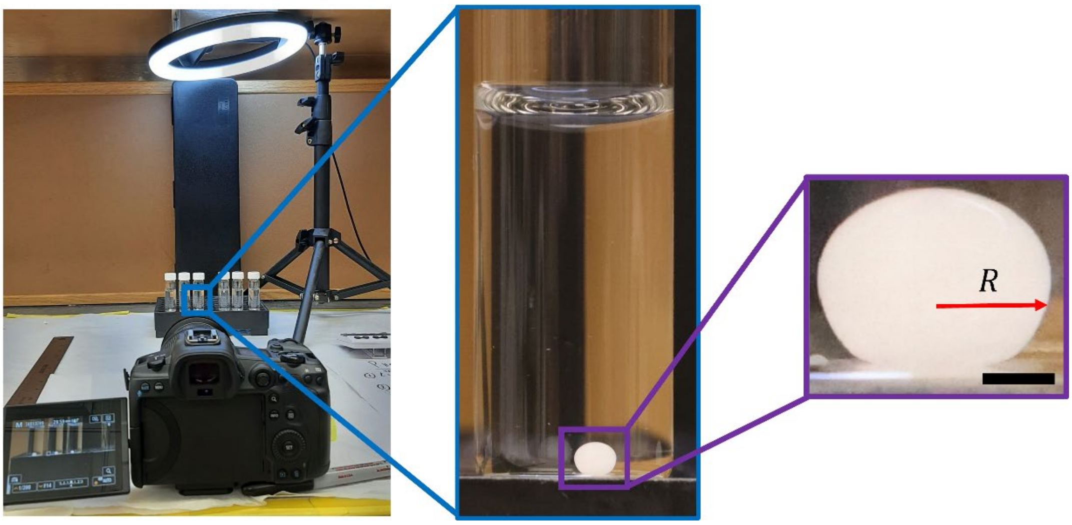

Supplementary Figure 4: Photo illustrating the imaging setup using the Canon EOS R5 digital camera to image the system under analysis with the scale bar indicating 1 mm. If the capillary length of the injected formulation ($\kappa{-}1$) is much larger than the radius of the droplet (R) under study, or equivalently if the Bond number is significantly smaller than 1 (Bo $\ll$ 1), gravity effects are negligible and the droplet will form a spherical cap on top of the glass substrate in order to minimize its surface energy. Based on this, the injected volume was selected to be 10 μL in such a way that the radius of the droplet was small enough to ensure that gravity and interfacial forces were of the same relative importance.

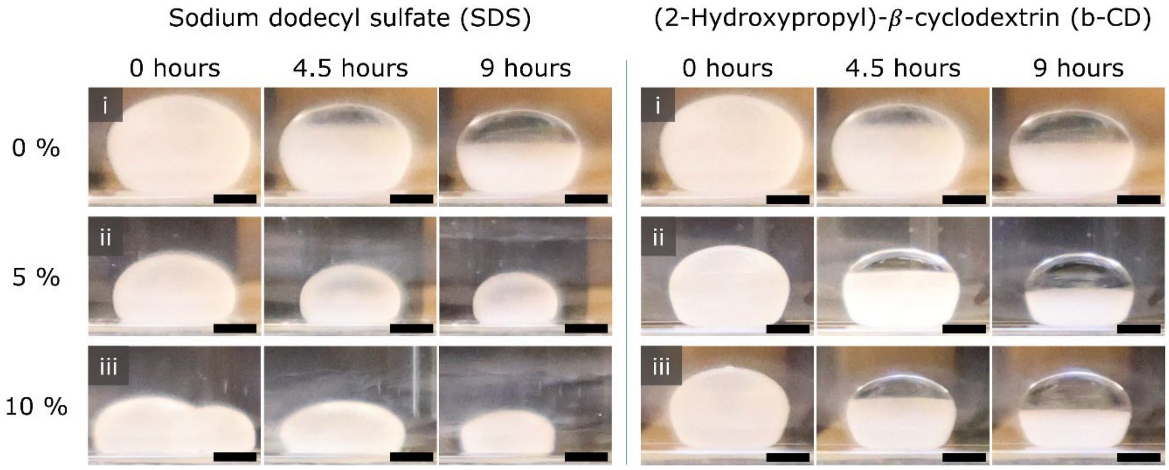

Supplementary Figure 5: Camera images showing effect of different kind of surfactants on depot formation kinetics from LNG/BB, specifically: sodium dodecyl sulfate (SDS) (left) and (2-Hydroxypropyl)-β-cyclodextrin (b-CD) (right), and different surfactant concentrations, i) 0%, ii) 5% and iii) 10%. Scale bars are 1mm.

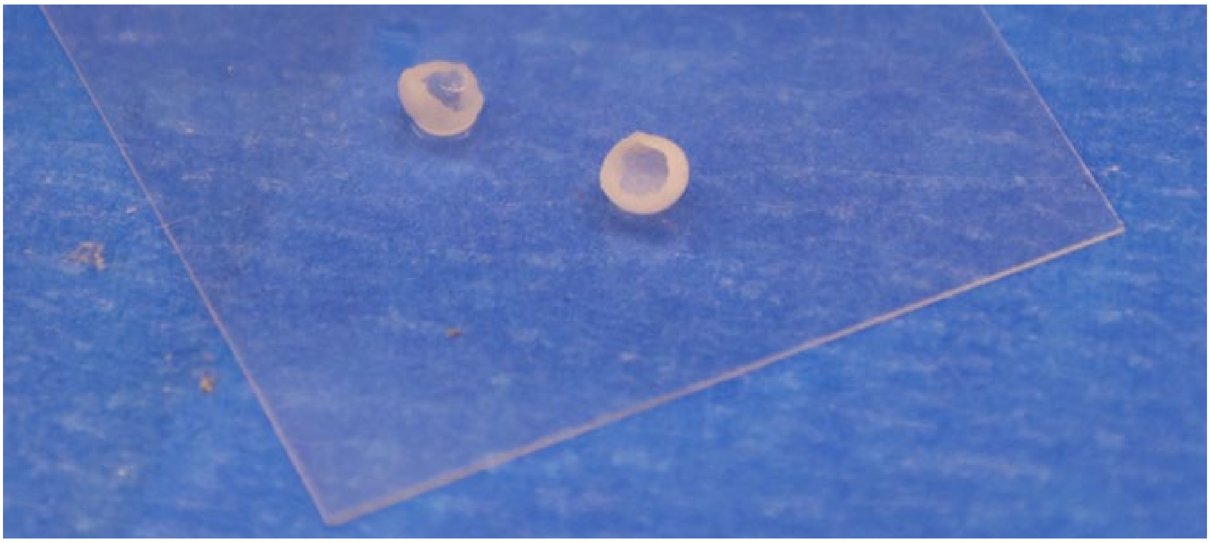

Supplementary Figure 6: Depots assembled from BA/LNG (50 mg/mL) in PBS highlighting the inner concavity which was observed both under the SEM as well as by visual inspection. In this image, the depots are being dried on top of a standard glass cover slip (50 x 24mm).

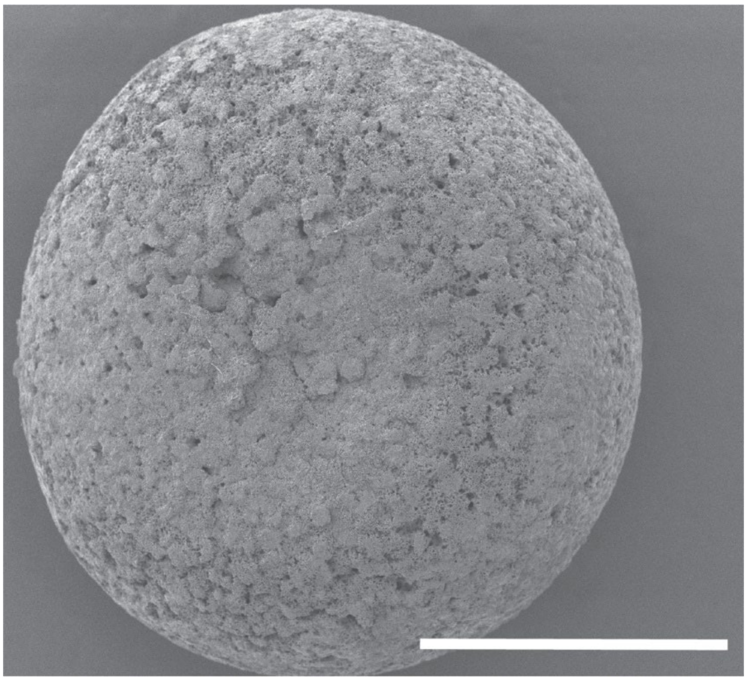

Supplementary Figure 7: Scanning electron microscopy image showing the compact spherical depot that was observed after solvent exchange of 50 mg/mL LNG in BB with PCL in PBS/10 wt% SDS. Scale bar is 500 µm.

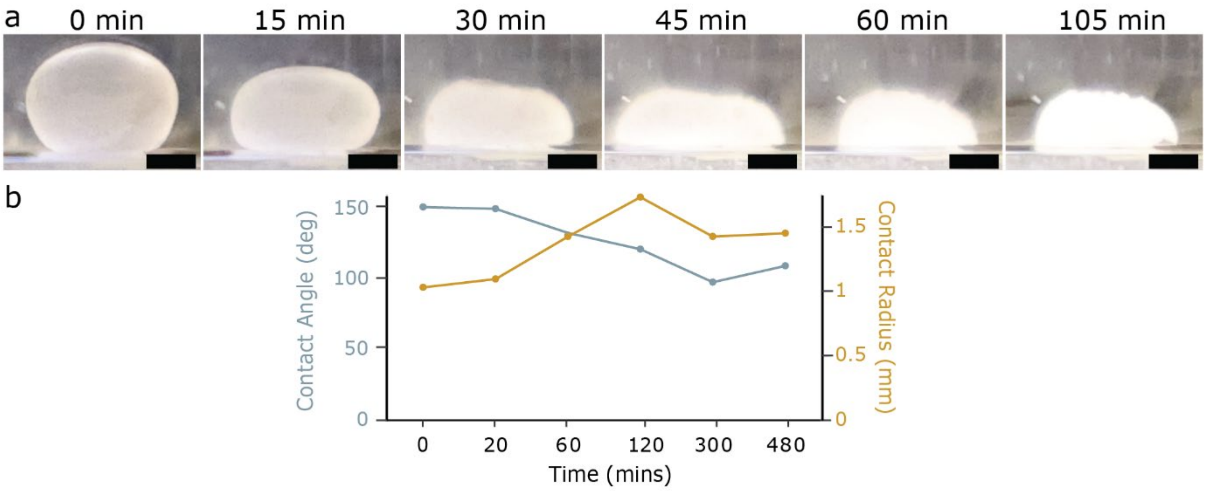

Supplementary Figure 8: (**a**) Time lapse images of 50 mg/mL LNG with BA in PBS. **(b)** After around 60 minutes, the contact angle decreases, while the contact radius ceases to decrease, illustrating that there is self-pinning. Scale bars are 1 mm.

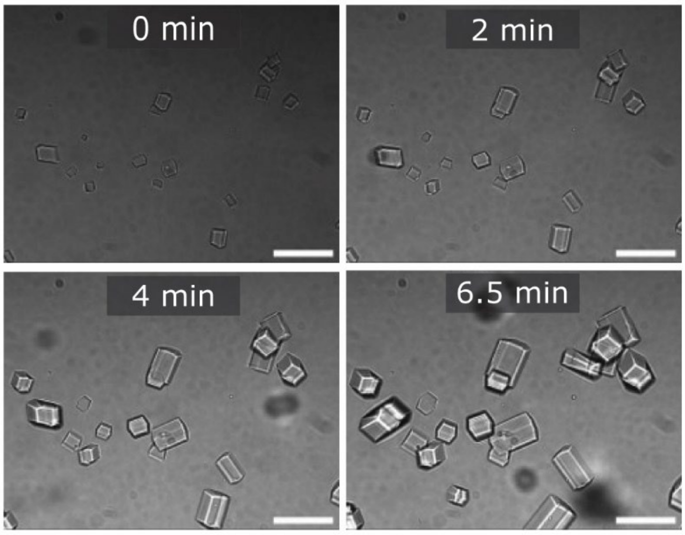

Supplementary Figure 9: Timelapse of LNG secondary nucleation and crystal growth from BA/LNG formulation exchanging solvent in pure PBS medium for 0, 2, 4 and 6.5 minutes. The system was imaged using a Zeiss LSM 980 confocal microscope with Zeiss AxioObserver Z1 stand and camera (Zeiss Axiocam 305 mono, CMOS camera) in brightfield mode. Scale bar is 30 µm.

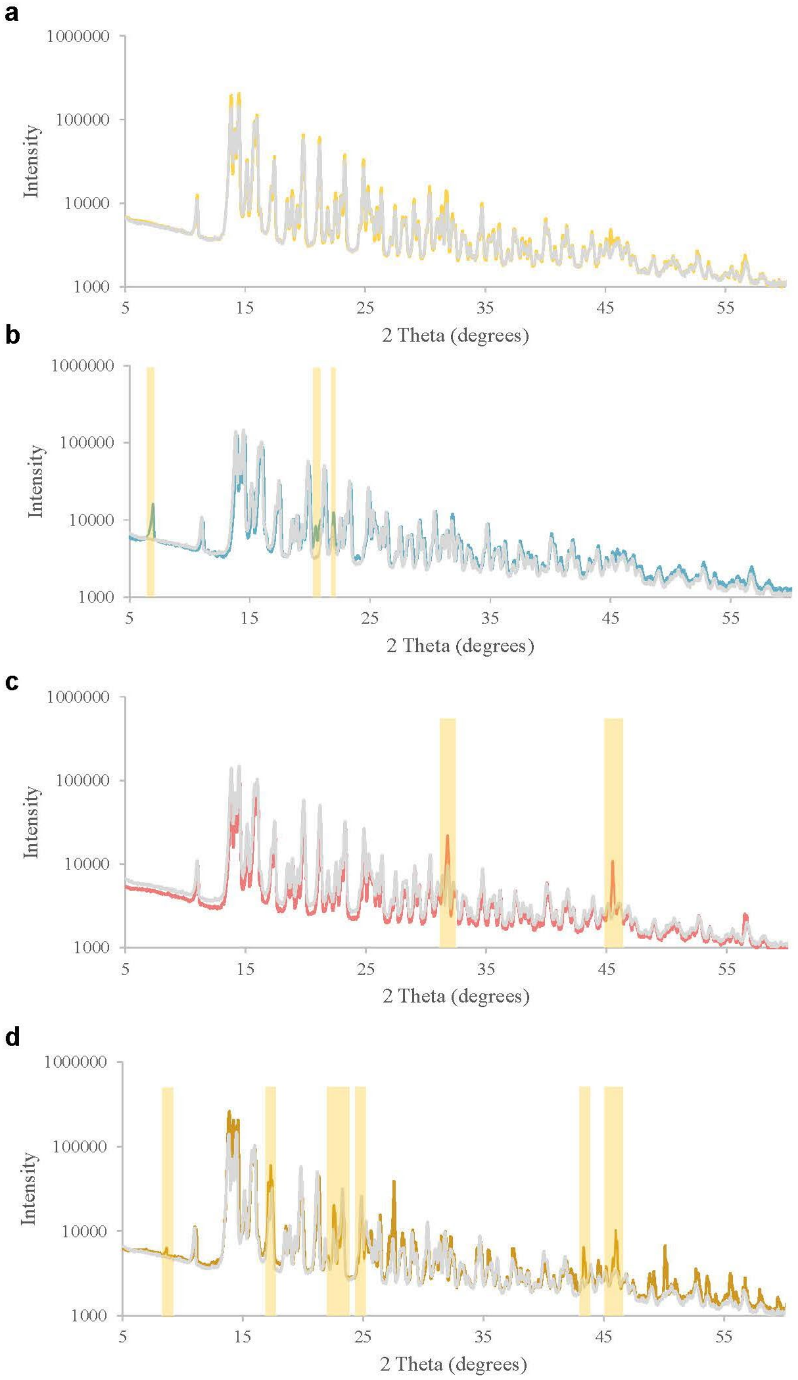

Supplementary Figure 10: Visual representations of various powder XRD spectra for different samples compared to as-supplied LNG powder control (grey). (**a**) After solvent exchange between suspensions of LNG in BA with PBS (yellow), the depot is identical to the LNG control. (**b**) The spectrum of the sample formed upon solvent exchange between LNG suspension in BB with PBS and 10% SDS (blue) overlaps well with the LNG powder control, but shows three additional peaks (highlighted in yellow). These can be attributed to the 1/8 hydrate form of SDS, whose crystal structure has been theoretically calculated by the Cambridge Crystallographic Data Center (CCDC)[33]. (c) After solvent exchange between suspensions of LNG in NMP with PBS (red), the resulting XRD spectrum is mostly identical to the LNG control, with two additional peaks (highlighted in yellow). (**d**) XRD spectrum of LNG precipitated from a solution of BA after solvent exchange in PBS (dark yellow), suggesting the formation of different crystalline polymorphs compared to the LNG control.

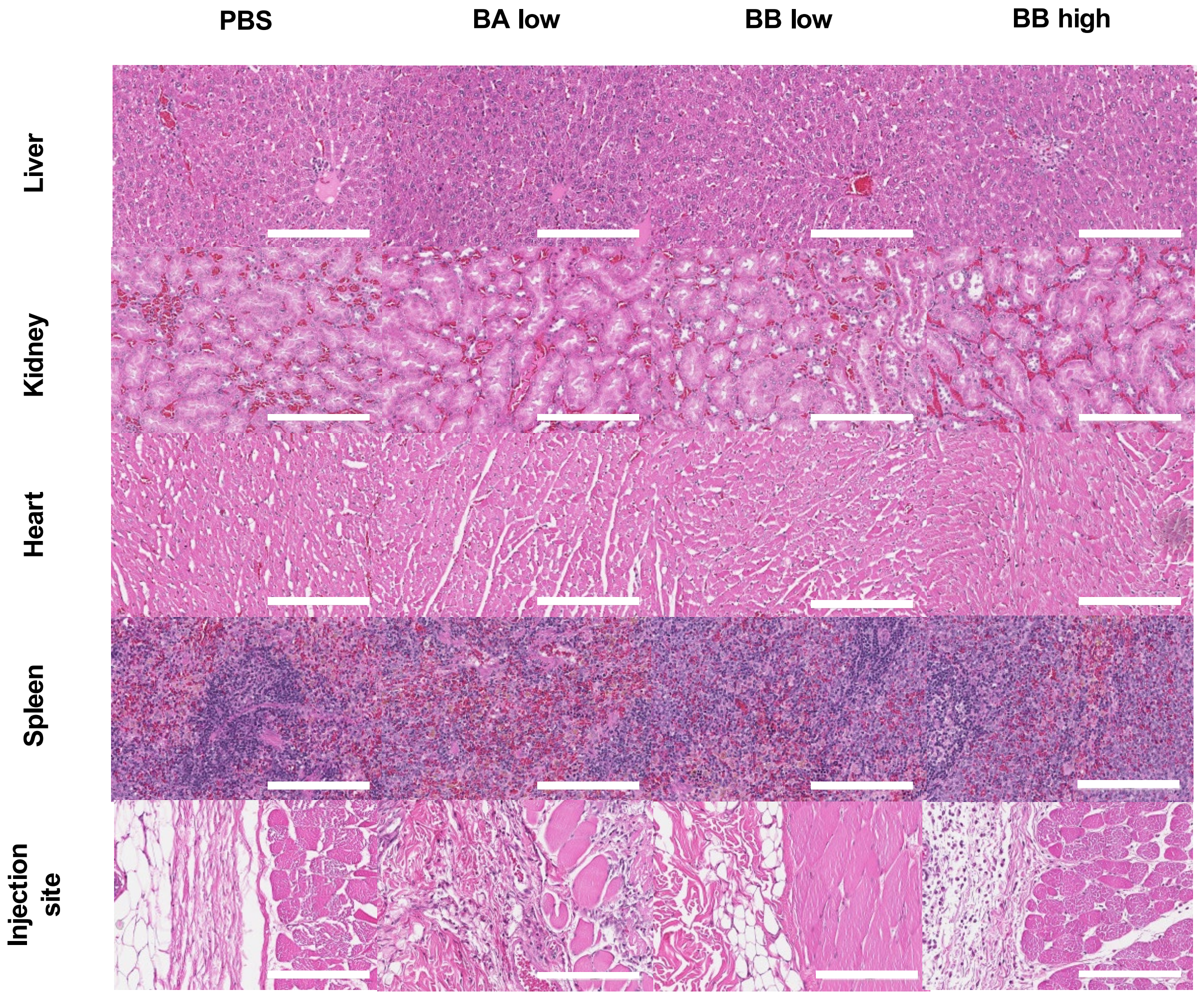

Supplementary Figure 11: Hematoxylin and eosin-stained organ tissues (liver, kidney, heart, and spleen) and injection sites from rats on Day 3, injected with PBS, BA at a low dose (240 µL/kg body weight), BB at a low dose (240 µL/kg body weight), and BB at a high dose (1600 µL/kg body weight). Scale bar is 200 µm.

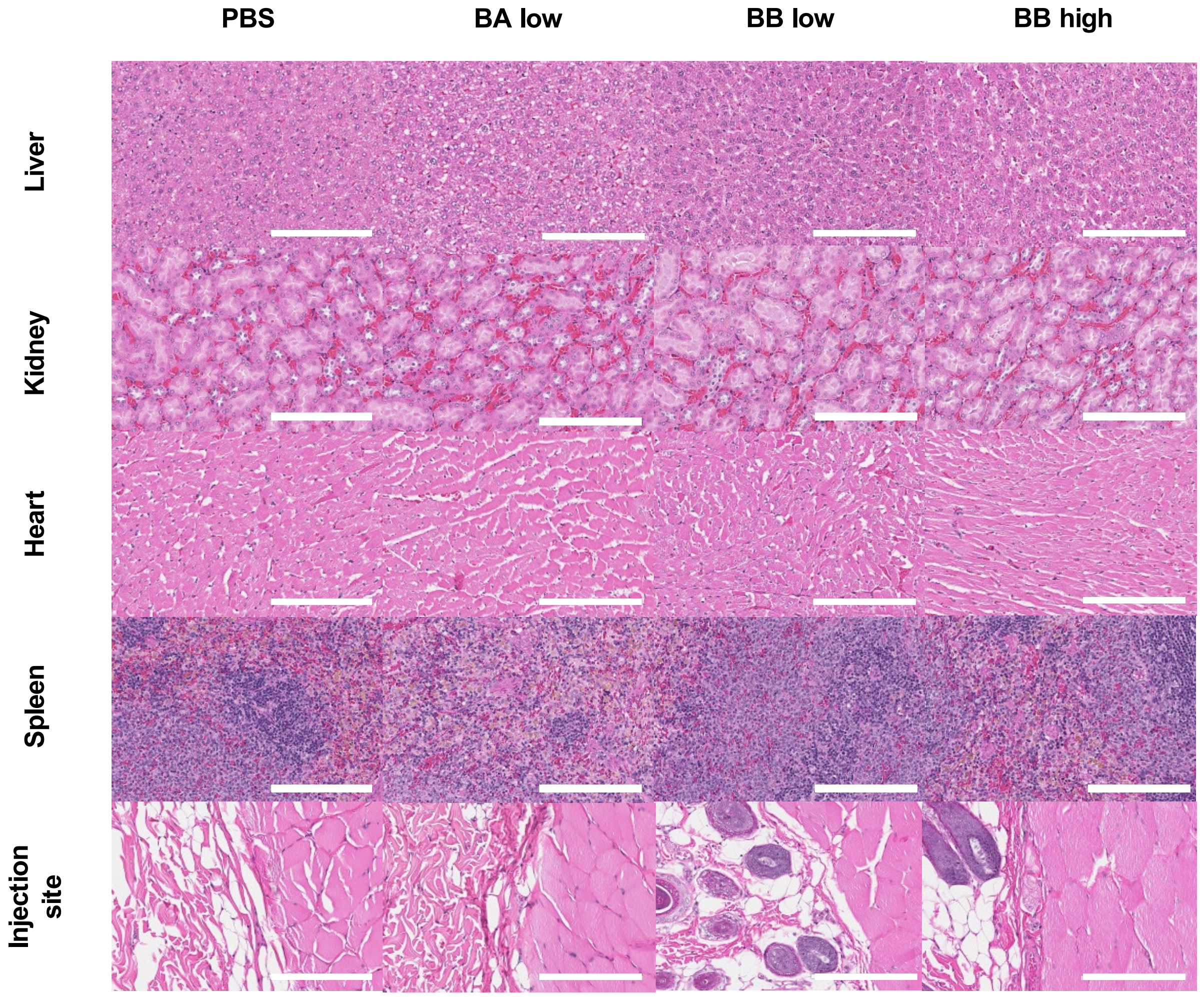

Supplementary Figure 12: Hematoxylin and eosin-stained organ tissues (liver, kidney, heart, and spleen) and injection sites from rats on Day 28, injected with PBS, BA at a low dose (240 µL/kg body weight), BB at a low dose (240 µL/kg body weight), and BB at a high dose (1600 µL/kg body weight). Scale bar is 200 µm.

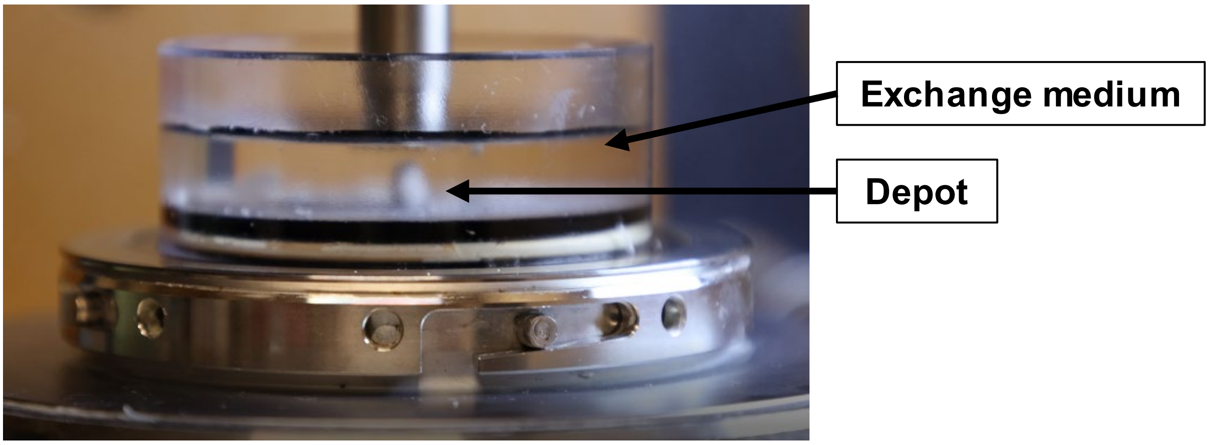

Supplementary Figure 13: Rheometer setup with solvent trap while performing a compressive study of a depot on the Discovery Hybrid Rheometer 3.

Solvent	LNG solubility (mg/ml) (n=3)	
	Mean	SD
PBS	Insoluble	
NMP	109.84	21.78
BA	28.05	5.15
BB	5.97	2.22

Supplementary Figure 14: Saturation concentration of LNG in solvents

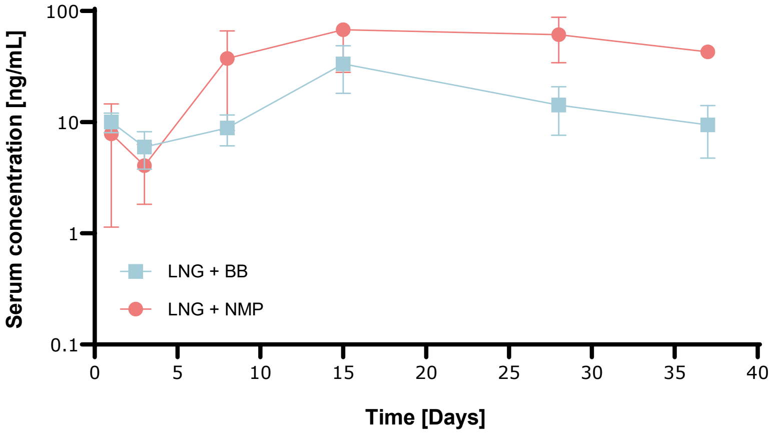

Supplementary Figure 15: Pharmacokinetic plot over 37 days from rodents injected LNG/BB and LNG/NMP formulation (n=3 each) subcutaneously. The NMP formulation displayed a significantly enhanced absorption profile. The area under the curve (AUC), over a 37-day period for the NMP formulation's AUC was approximately 2.9 times higher than for BB, at 1790 ± 383.4 ng/ml·day, in contrast to the BB formulation, at 617.7 ± 126.8 ng/ml·day. n =3 per group. Data represent mean + s.d.

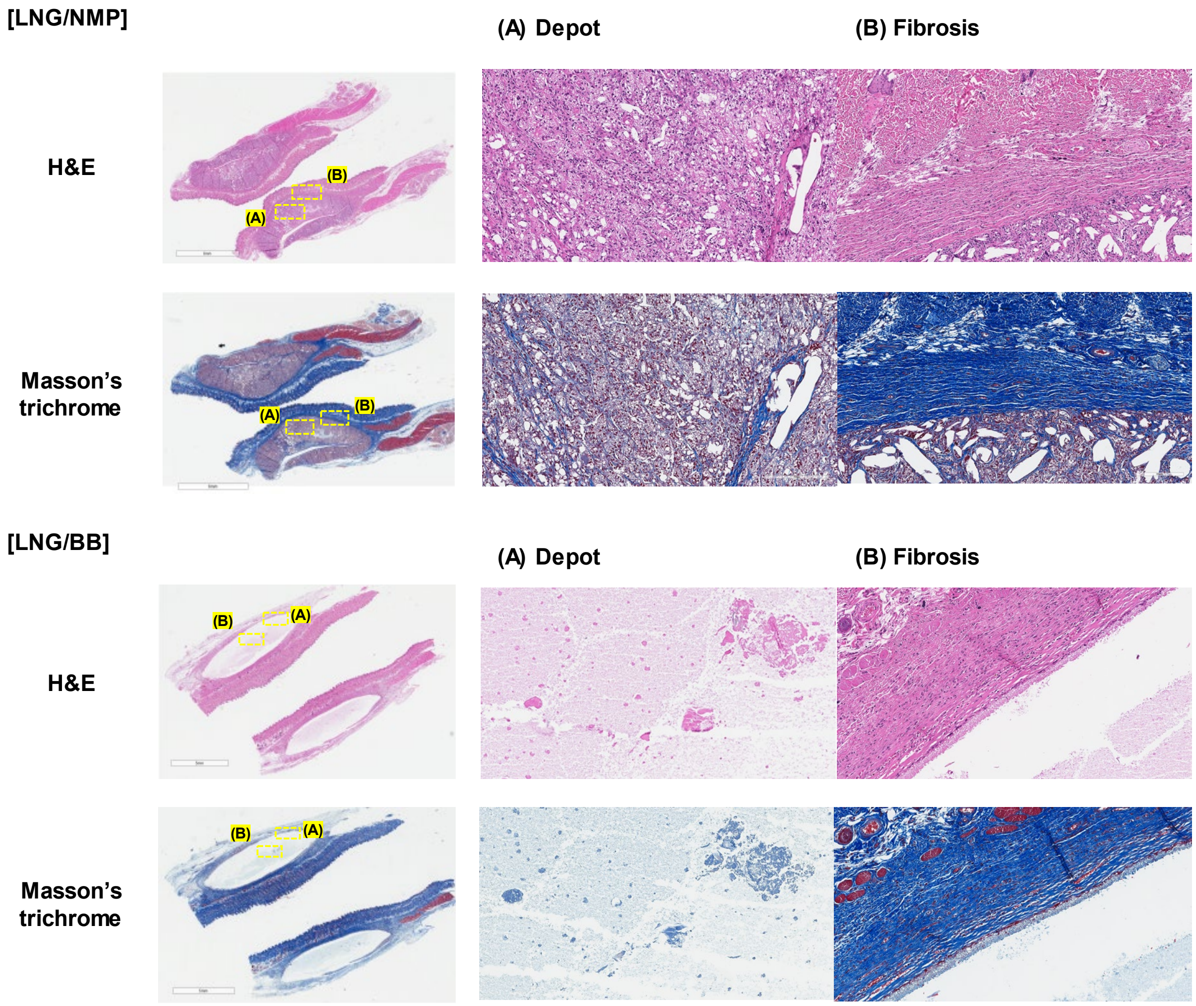

Supplementary Figure 16: Histopathological analysis of LNG/BB and LNG/NMP formulations on Day 37 using hematoxylin and eosin (H&E) and Masson's trichrome (MT) stains shows that the LNG/NMP formulation induces moderate inflammation and extensive scar tissue formation, while the LNG/BB formulation exhibits minimal inflammation and fibrosis, indicating better biocompatibility and safety. Cross-section of the implant shows the NMP formulation with significant scar tissue and macrophage infiltration, indicating inflammation, while the BB formulation exhibits minimal fibrosis and inflammation, demonstrating its enhanced biocompatibility and safety.

Supplementary Note 2 | Pharmacokinetic analysis and drug retention insights from a 97-day in vivo rodent study

At the end of our 97-day in vivo study, LNG still remained at the injection site for all 3 groups. Based on the differences between the groups, we can surmise that the formulations comprising BB should enable substantially longer release durations compared to the control group in PBS. To quantify this benefit, we performed a ballpark estimate of the total remaining drug after 97 days.

Key Assumptions:

1. Area under the curve (AUC) Represents Total Exposure Over Time: The AUC values represent cumulative drug exposure over the 97-day period. We assume the AUC values are accurate and reflective of the entire duration.
2. Clearance (Cl) is Constant: The clearance value (1.45 L/hr or 34.8 L/day) is assumed to be constant throughout the study period. This value is derived from the work by Ko et al. (2022) on the pharmacokinetics of levonorgestrel in rodents.
3. Complete Absorption: We assume that the entire amount of drug released from the implant is absorbed into systemic circulation and reflected in the AUC.

PK Data and Clearance Assumption:

- The Area Under the Curve (AUC) for each formulation over the 97-day period is as follows:
    - LNG/PBS: 3401 ng/mL·day
    - LNG/BB: 1537 ng/mL·day
    - LNG/BB+PCL: 605.3 ng/mL·day
- The clearance value (Cl) is assumed to be 1.45 L/hr, or 34.8 L/day, based on Ko et al. (2022) [34].

Total Drug Released:
Based on these assumptions, we calculate the total drug released as follows:

- For LNG/PBS, the total drug released is approximately 118.35mg.
- For LNG/BB, the total drug released is approximately 53.52mg.
- For LNG/BB+PCL, the total drug released is approximately 21.07mg.

Remaining Drug Estimate:
Given the initial loading of LNG in the implant (146.5 mg), the estimated amount of drug remaining in the implant after 97 days is:

- For LNG/PBS, approximately 28.15mg remains.
- For LNG/BB, approximately 92.98mg remains.
- For LNG/BB+PCL, approximately 125.43mg remains.

The results indicate that the LNG/BB formulation performs significantly better than LNG/PBS in retaining the drug within the implant, with approximately 63.5% of the initial drug loading remaining after 97 days, compared to only 19.2% for LNG/PBS. This improvement underscores the efficacy of the compacted form enabled by benzyl benzoate as a vehicle. Furthermore, the LNG/BB+PCL formulation demonstrates that incorporating a small amount of polymer can further fine-tune the release profile, retaining as much as 85.6% of the drug. This tunability highlights the potential of such systems to achieve a balance between release duration and sustained drug levels, making it adaptable for diverse therapeutic needs.

This estimated comparison is conservative because of the observed differences in porosity via our microCT analysis, which should amplify the differences in release kinetics over longer time periods: the porous LNG/PBS formulation is expected to continue to release drug via bulk erosion, whereas the highly densified BB formulations are expected to primarily release drug via surface erosion.

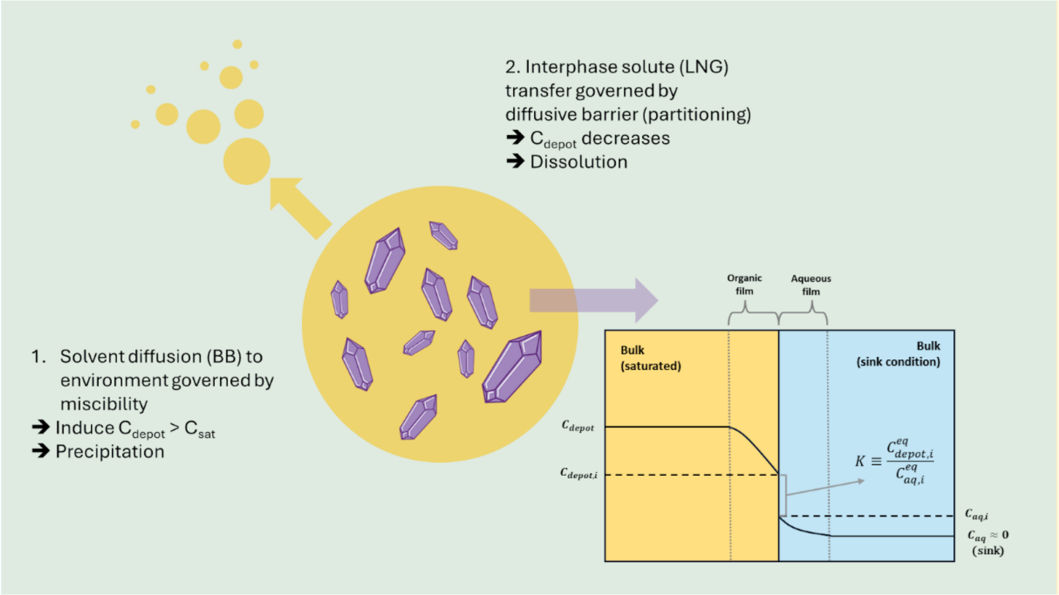

Supplementary Figure 17: Schematic representation of competing solvent and solute diffusion mechanisms in LNG depot dynamics

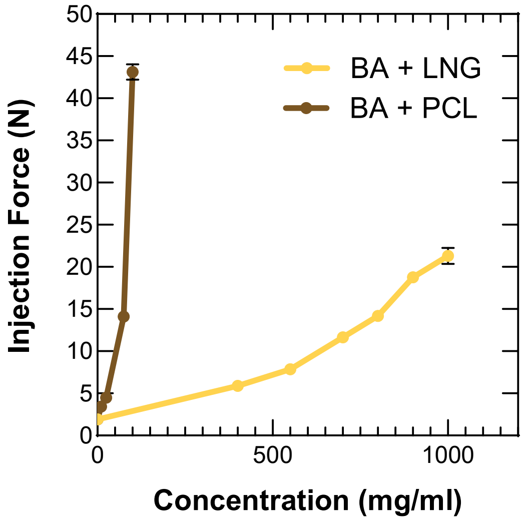

Supplementary Figure 18: Effect of increasing concentration of LNG microcrystals (yellow) versus solubilized PCL (brown) on the injection force required to extrude the suspension and solution, respectively, through a 23G needle at 6 mL/min. n =3 per group. Data represent mean + s.d.

References

1. Young, I. C. *et al.* Long-acting injectable multipurpose prevention technology for prevention of HIV and unplanned pregnancy. *Journal of Controlled Release* **363**, 606–620 (2023).
2. Tang, J. *et al.* Coaxial electrospray of uniform polylactide core-shell microparticles for long-acting contraceptive. *Journal of Controlled Release* **341**, 634–645 (2022).
3. Janagam, D. R. *et al.* Injectable In Situ Forming Depot Systems for Long-Acting Contraception. *Advanced Biosystems* **1**,.
4. Zhang, X. *et al.* Feasibility of poly (**ε** -caprolactone-co-DL-lactide) as a biodegradable material for *in situ* forming implants: evaluation of drug release and *in vivo* degradation. *Drug Development and Industrial Pharmacy* **41**, 342–352 (2015).
5. Puthli, S. & Vavia, P. Formulation and Performance Characterization of Radio-Sterilized "Progestin-Only" Microparticles Intended for Contraception. *AAPS PharmSciTech* **10**, 443–452 (2009).
6. Sun, Y. *et al.* Synchronic release of two hormonal contraceptives for about one month from the PLGA microspheres: in vitro and in vivo studies. *Journal of Controlled Release* **129**, 192–199 (2008).
7. Wang, S. H. *et al.* Controlled release of levonorgestrel from biodegradable poly (D, L-lactide-co-glycolide) microspheres: in vitro and in vivo studies. *International journal of pharmaceutics* **301**, 217–225 (2005).
8. Dhanaraju, M. D., RajKannan, R., Selvaraj, D., Jayakumar, R. & Vamsadhara, C. Biodegradation and biocompatibility of contraceptive-steroid-loaded poly (DL-lactide-co-glycolide) injectable microspheres: in vitro and in vivo study. *Contraception* **74**, 148–156 (2006).
9. Pfizer. SAYANA PRESS. https://labeling.pfizer.com/ShowLabeling.aspx?id=15014 (2022).
10. Food and Drug Administration. DEPO-SUBQ PROVERA 104. https://www.accessdata.fda.gov/drugsatfda_docs/label/2019/021583s016,%20s024,%20s029lbl.pdf (2019).

11. Food and Drug Administration. DEPO-PROVERA CI. https://www.accessdata.fda.gov/drugsatfda_docs/label/2010/020246s036lbl.pdf.
12. Nakhla, D. S. *et al.* Injectable long-acting ivacaftor-loaded poly (lactide-co-glycolide) microparticle formulations for the treatment of cystic fibrosis: In vitro characterization and in vivo pharmacokinetics in mice. *International journal of pharmaceutics* **650**, 123693 (2024).
13. Young, I. C. *et al.* Ultra-long-acting in-situ forming implants with cabotegravir protect female macaques against rectal SHIV infection. *Nat Commun* **14**, 708 (2023).
14. Wang, S. *et al.* Aqueous remote loading of setmelanotide in poly (lactic-co-glycolic acid) microspheres for long-term obesity treatment. *Journal of Controlled Release* **364**, 589–600 (2023).
15. Zhao, D. *et al.* Long-acting injectable in situ gel of rasagiline: a patented product development. *Drug Deliv. and Transl. Res.* **13**, 1012–1021 (2023).
16. Rajadhyaksha, P. M., Shastri, D. H. & Shah, D. K. Pharmacokinetic Evaluation of Thermosensitive Sustained Release Formulations Developed for Subcutaneous Delivery of Protein Therapeutics. *Journal of Pharmaceutical Sciences* **112**, 868–876 (2023).
17. Wu, X. *et al.* A liquid crystal in situ gel based on rotigotine for the treatment of Parkinson's disease. *Drug Deliv. and Transl. Res.* **14**, 1048–1062 (2024).
18. Kim, M. *et al.* New long-acting injectable microspheres prepared by IVL-DrugFluidic™ system: 1-month and 3-month in vivo drug delivery of leuprolide. *International journal of pharmaceutics* **622**, 121875 (2022).
19. Kim, M. *et al.* A long-acting formulation of rifabutin is effective for prevention and treatment of Mycobacterium tuberculosis. *Nat Commun* **13**, 4455 (2022).
20. Choi, G.-W., Lee, S., Kang, D. W., Kim, J. H. & Cho, H.-Y. Long-acting injectable donepezil microspheres: Formulation development and evaluation. *Journal of Controlled Release* **340**, 72–86 (2021).
21. Zhai, J. *et al.* Exenatide-loaded inside-porous poly(lactic-co-glycolic acid) microspheres as a long-acting drug delivery system with improved release characteristics. *Drug Delivery* **27**, 1667–1675 (2020).

22. Benhabbour, S. R. *et al.* Ultra-long-acting tunable biodegradable and removable controlled release implants for drug delivery. *Nat Commun* **10**, 4324 (2019).
23. Food and Drug Administration. BRIXADI. https://www.accessdata.fda.gov/drugsatfda_docs/label/2023/210136Orig1s000lbl.pdf (2023).
24. Food and Drug Administration. SUBLOCADE. https://www.accessdata.fda.gov/drugsatfda_docs/label/2017/209819s000lbl.pdf (2017).
25. Food and Drug Administration. CAMCEVI. https://www.accessdata.fda.gov/drugsatfda_docs/label/2021/211488s000lbl.pdf (2021).
26. Food and Drug Administration. FENSOLVI. https://www.accessdata.fda.gov/drugsatfda_docs/label/2020/213150s000lbl.pdf (2020).
27. Food and Drug Administration. BYDUREON. https://www.accessdata.fda.gov/drugsatfda_docs/label/2018/022200s026lbl.pdf (2018).
28. Food and Drug Administration. PERSERIS. https://www.accessdata.fda.gov/drugsatfda_docs/label/2018/210655s000lbl.pdf (2018).
29. Food and Drug Administration. ELIGARD. https://www.accessdata.fda.gov/drugsatfda_docs/label/2016/021343s033,021379s033,021488s030,021731s029lbl.pdf (2016).
30. Food and Drug Administration. Norditropin. https://www.accessdata.fda.gov/drugsatfda_docs/label/2017/021148s049lbl.pdf (2017).
31. Food and Drug Administration. SUSTOL. https://www.accessdata.fda.gov/drugsatfda_docs/label/2016/022445s000lbl.pdf (2016).
32. Food and Drug Administration. Nutropin AQ. (2014).
33. Lee, H. L., Cheng, Y. S., Yeh, K. L. & Lee, T. A Novel Hydrate Form of Sodium Dodecyl Sulfate and Its Crystallization Process. *ACS Omega* **6**, 15770–15781 (2021).
34. Ko, P. J., Milad, M. A., Radulovic, L. L. & Gibson, D. M. Pharmacokinetics of levonorgestrel and etonogestrel in rat or minipig following intravenous, subcutaneous, or intradermal administration. *Xenobiotica* **52**, 575–582 (2022).
