# Supplementary figures and images for: Self-aggregating long-acting injectable microcrystals
Source: Nat Chem Eng. 2025 Mar 24;2(3):209–19. doi: 10.1038/s44286-025-00194-x (PMC11932925; doi:10.1038/s44286-025-00194-x)

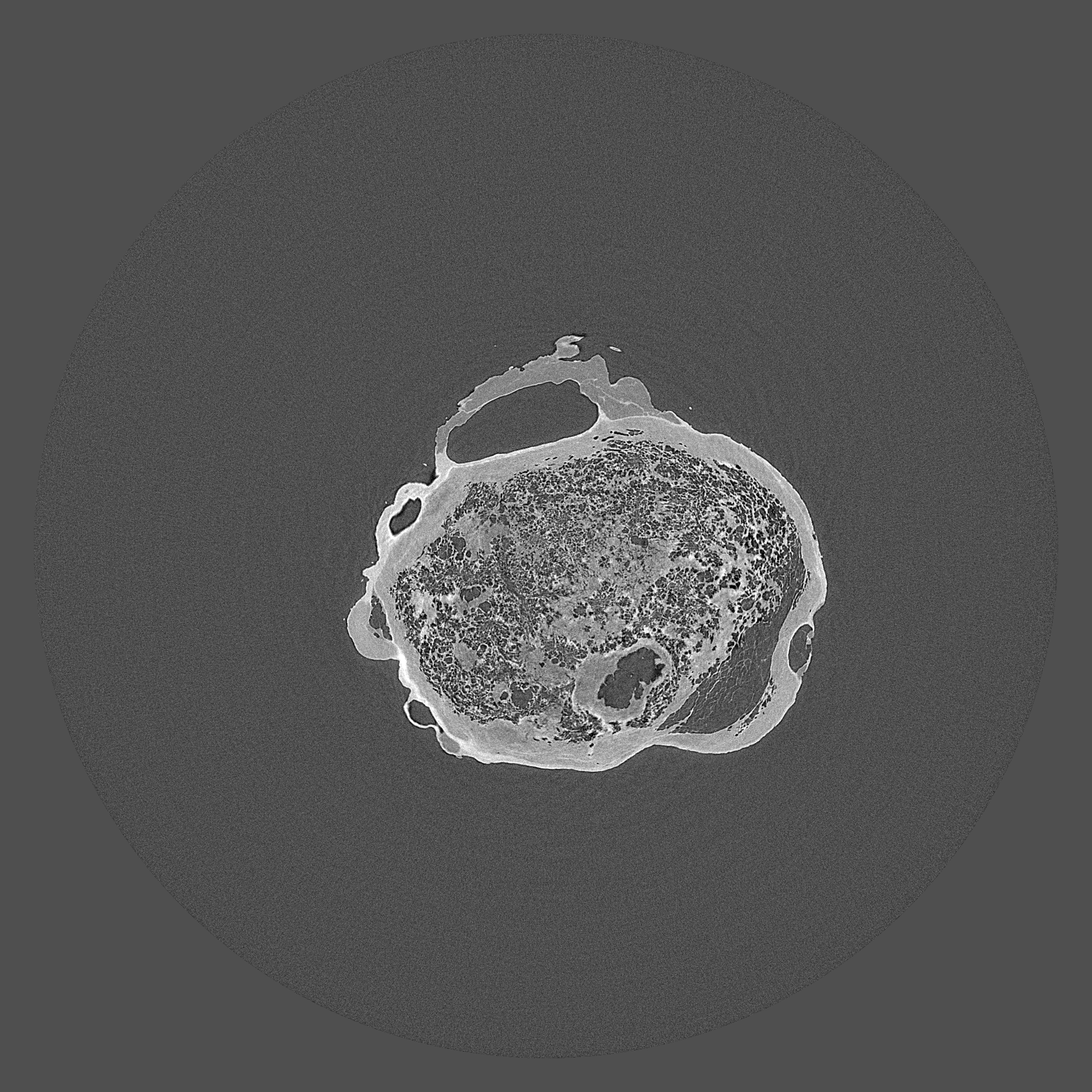

Supplement: Supplementary file 5 — μCT images. [file 44286_2025_194_MOESM5_ESM.zip › Souce Data - Figgure 5 C,D,E/C2_0900.tif]

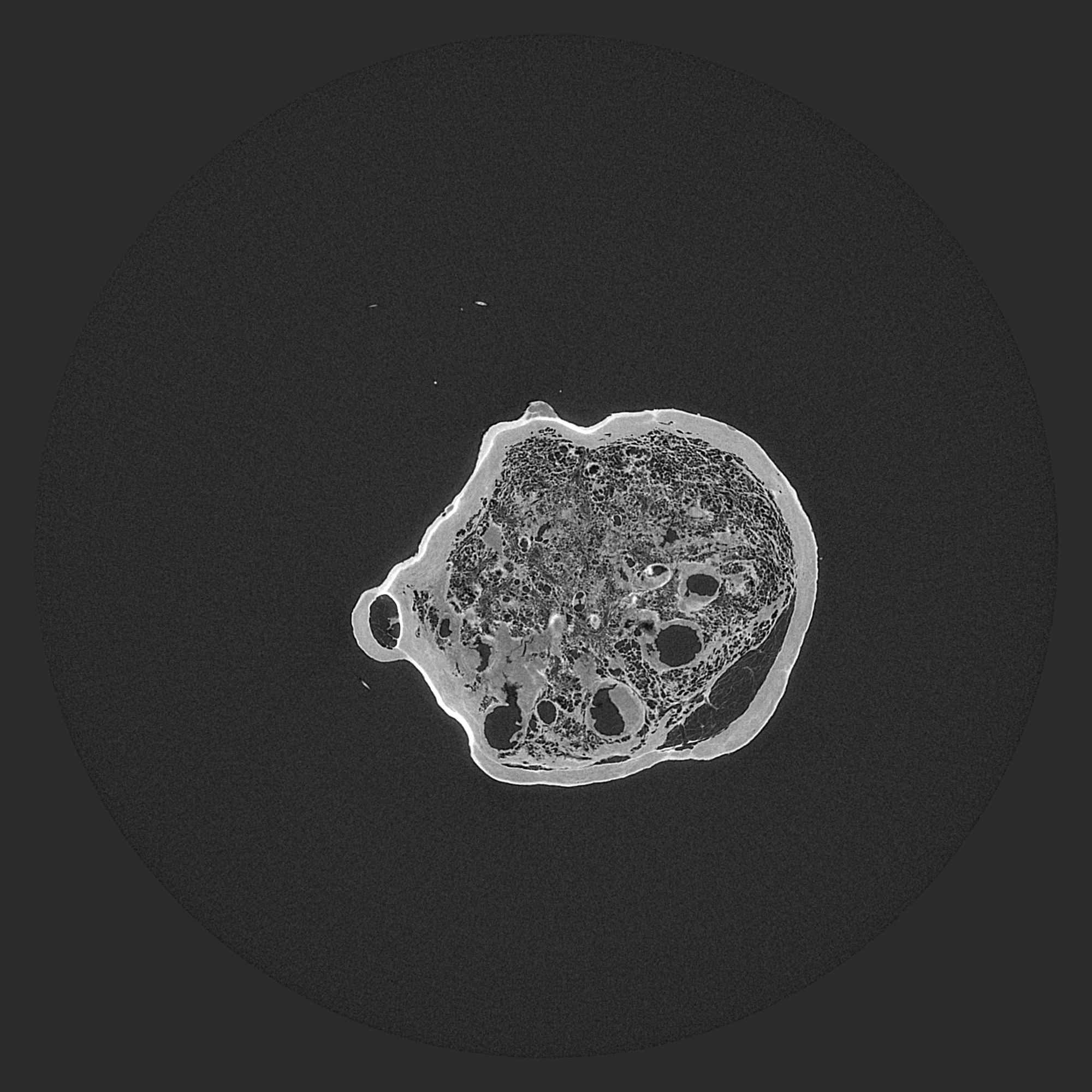

Supplement: Supplementary file 5 — μCT images. [file 44286_2025_194_MOESM5_ESM.zip › Souce Data - Figgure 5 C,D,E/C2_1000.tif]

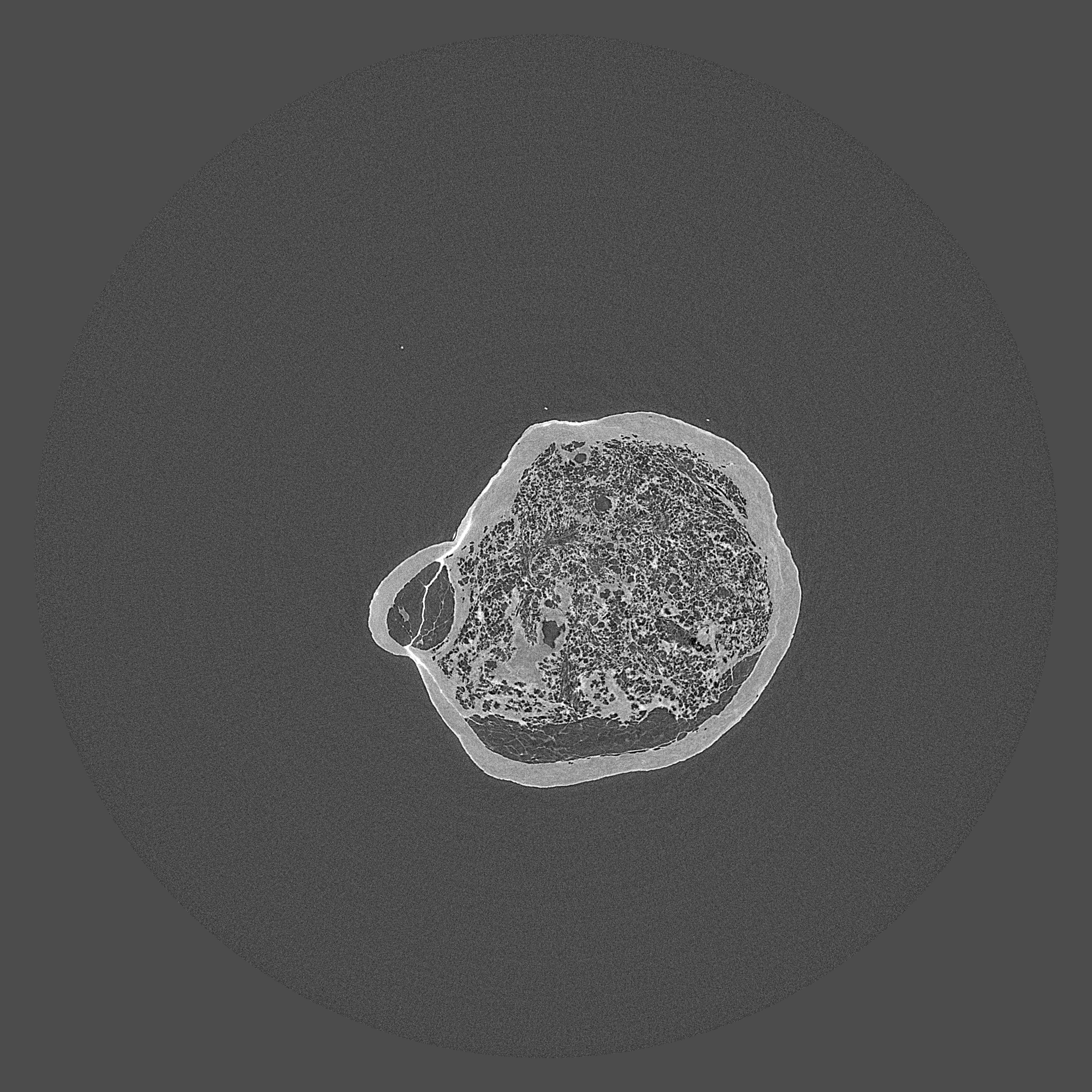

Supplement: Supplementary file 5 — μCT images. [file 44286_2025_194_MOESM5_ESM.zip › Souce Data - Figgure 5 C,D,E/C2_1100.tif]

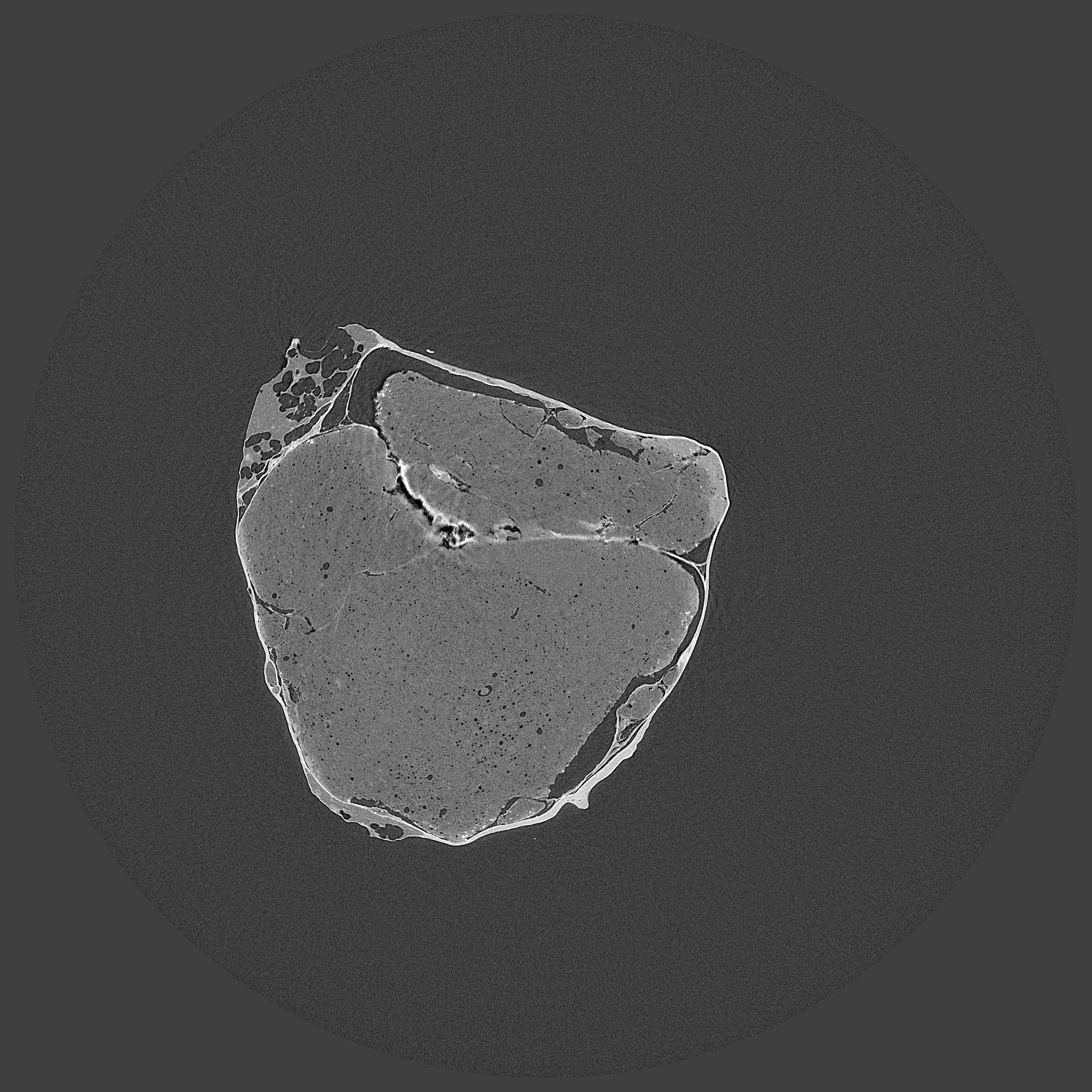

Supplement: Supplementary file 5 — μCT images. [file 44286_2025_194_MOESM5_ESM.zip › Souce Data - Figgure 5 C,D,E/D1_0900.tif]

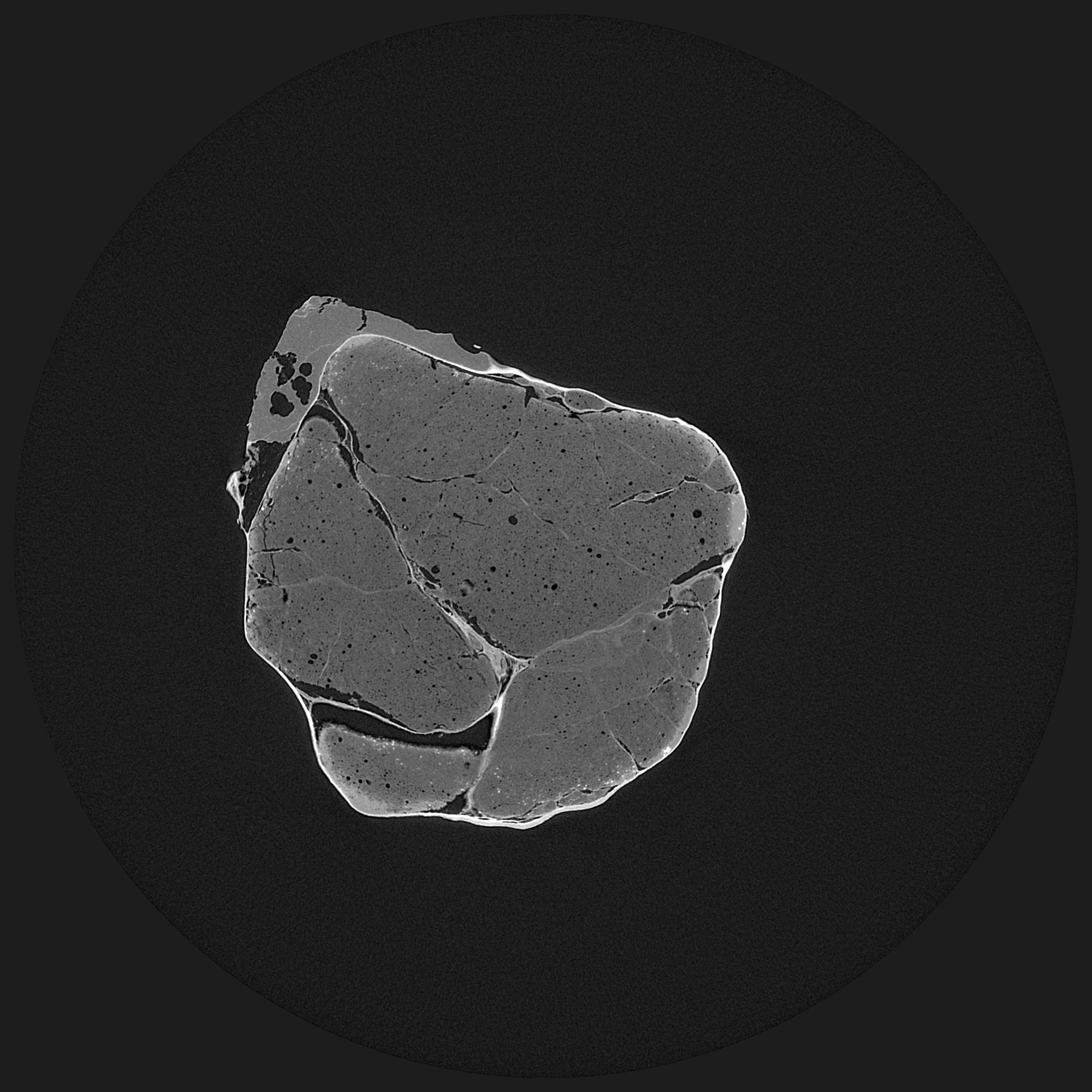

Supplement: Supplementary file 5 — μCT images. [file 44286_2025_194_MOESM5_ESM.zip › Souce Data - Figgure 5 C,D,E/D1_1000.tif]

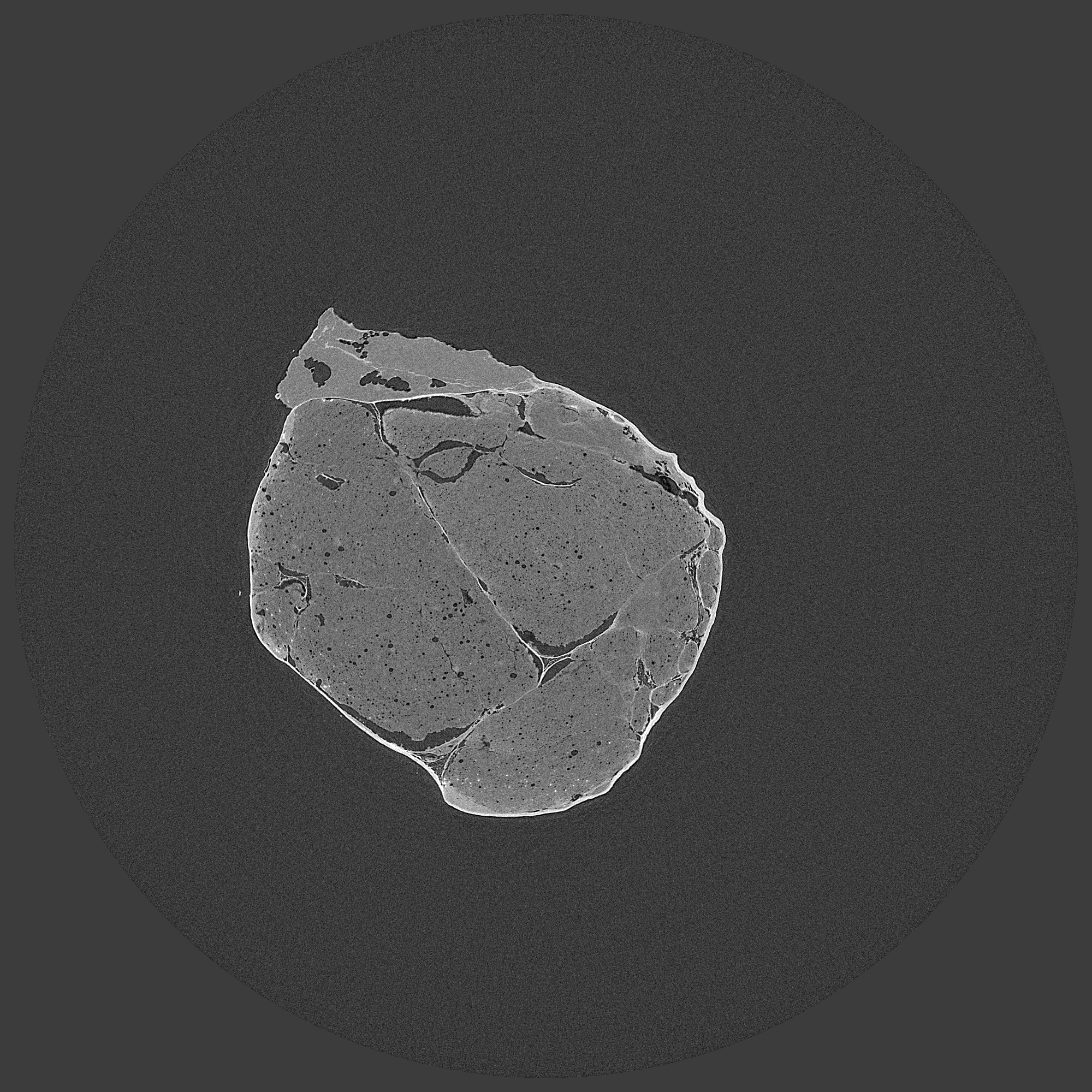

Supplement: Supplementary file 5 — μCT images. [file 44286_2025_194_MOESM5_ESM.zip › Souce Data - Figgure 5 C,D,E/D1_1100.tif]

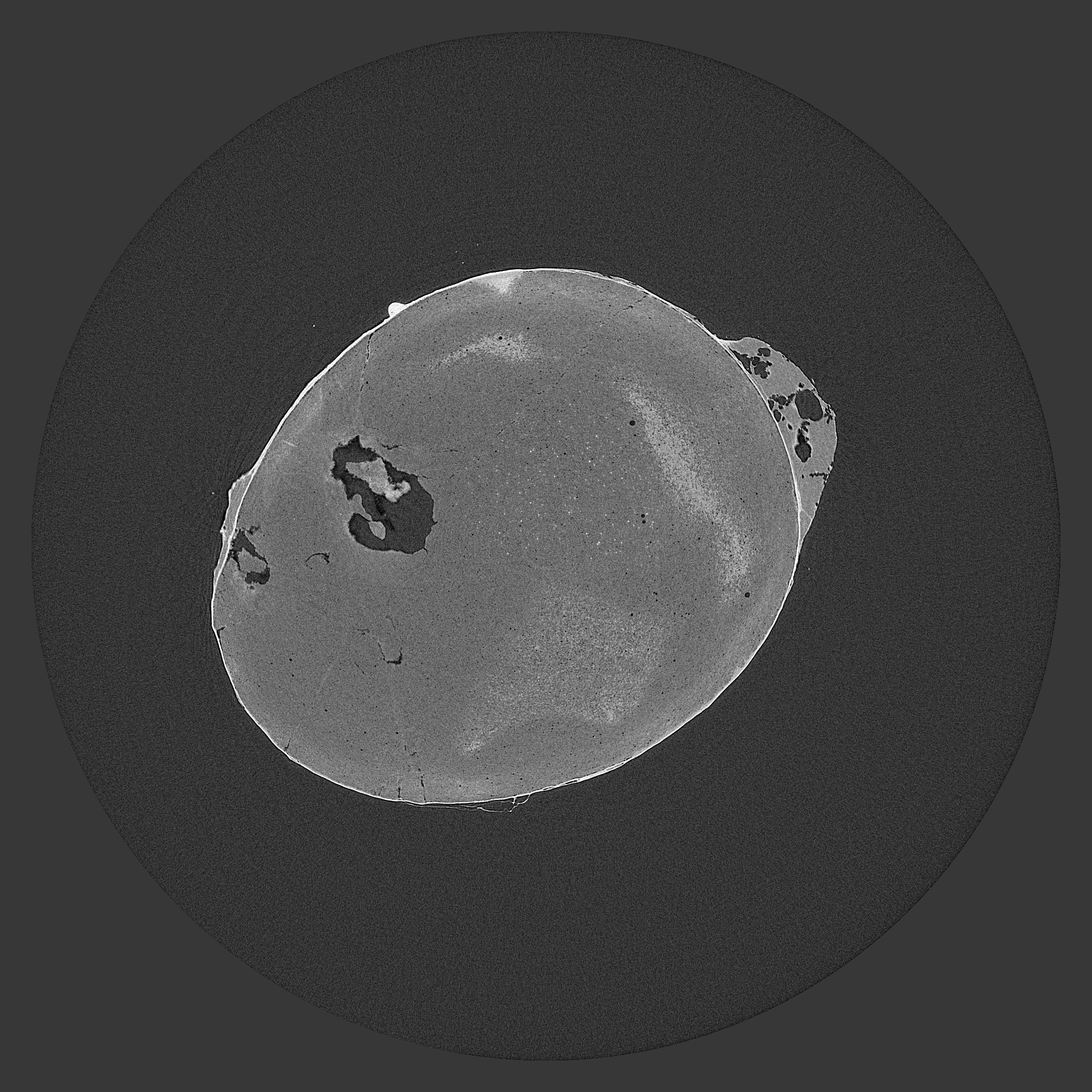

Supplement: Supplementary file 5 — μCT images. [file 44286_2025_194_MOESM5_ESM.zip › Souce Data - Figgure 5 C,D,E/E2_0900.tif]

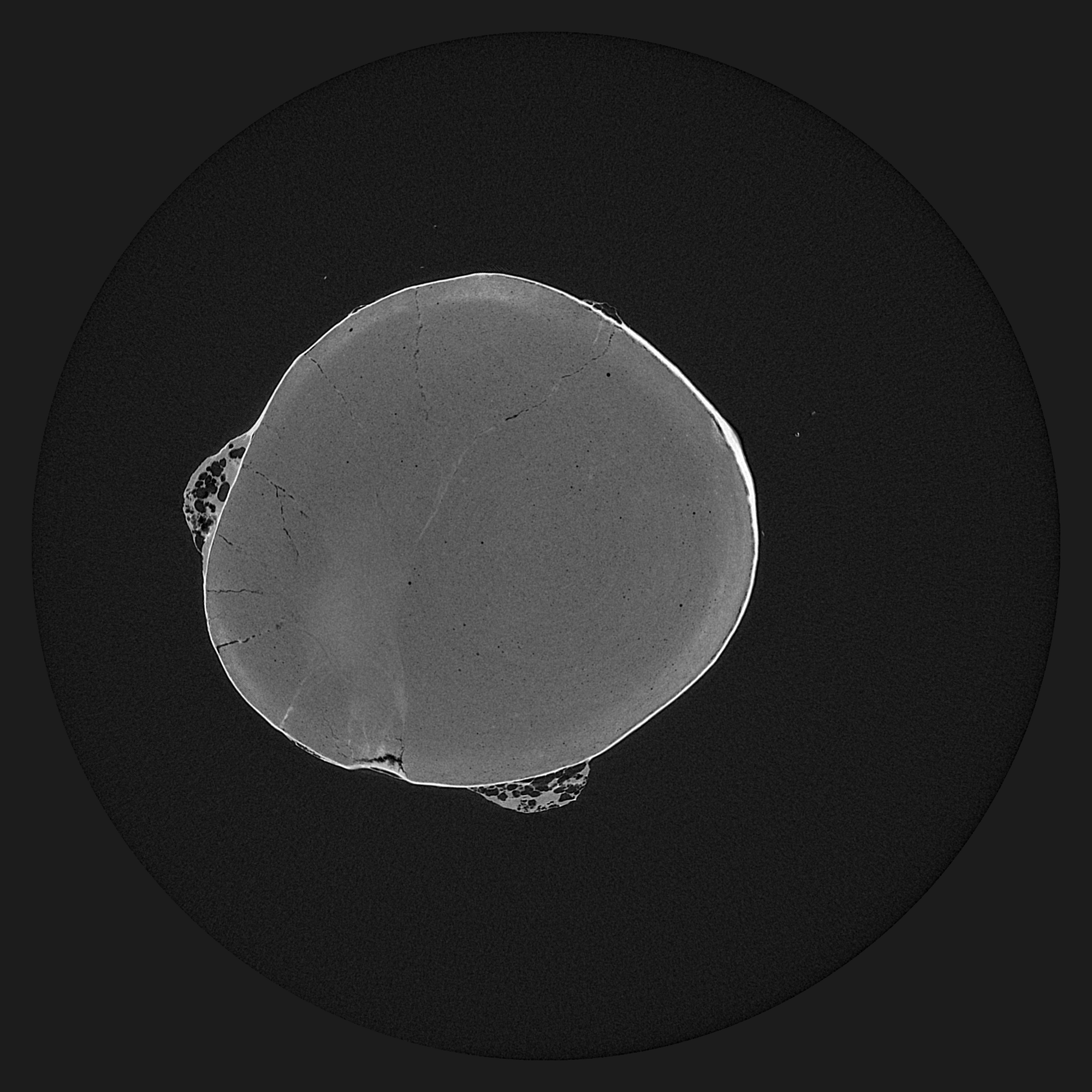

Supplement: Supplementary file 5 — μCT images. [file 44286_2025_194_MOESM5_ESM.zip › Souce Data - Figgure 5 C,D,E/E2_1000.tif]

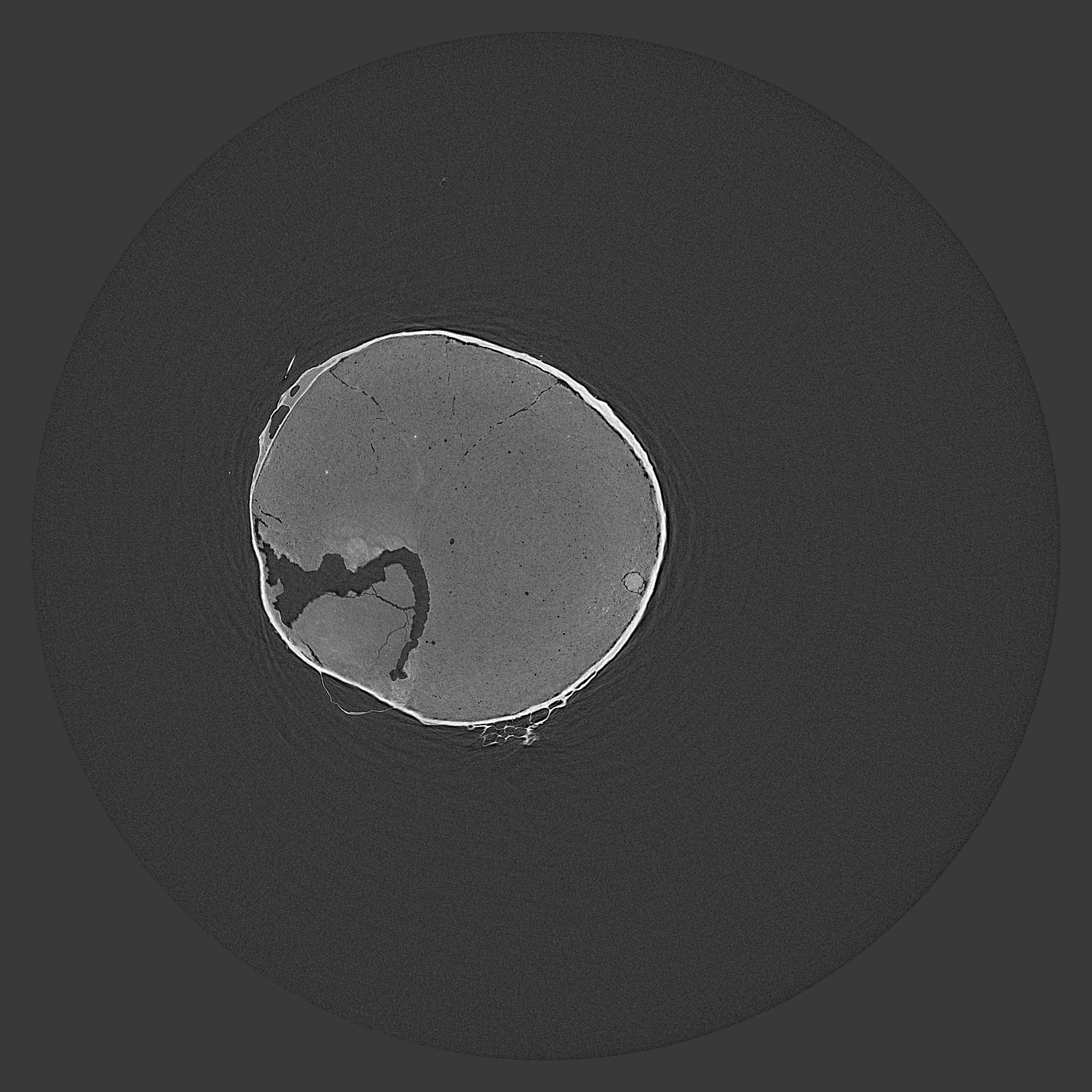

Supplement: Supplementary file 5 — μCT images. [file 44286_2025_194_MOESM5_ESM.zip › Souce Data - Figgure 5 C,D,E/E2_1100.tif]
